# Modelling grape growth in relation to whole-plant carbon and water fluxes

**DOI:** 10.1093/jxb/ery367

**Published:** 2018-10-23

**Authors:** Junqi Zhu, Michel Génard, Stefano Poni, Gregory A Gambetta, Philippe Vivin, Gilles Vercambre, Michael C T Trought, Nathalie Ollat, Serge Delrot, Zhanwu Dai

**Affiliations:** 1 EGFV, Bordeaux Sciences Agro, INRA, Université de Bordeaux, ISVV, Villenave d’Ornon, France; 2 The New Zealand Institute for Plant and Food Research Limited (PFR) Marlborough, Blenheim, New Zealand; 3 INRA, UR 1115 Plantes et Systèmes de Culture Horticoles, Avignon, France; 4 Department of Sustainable Crop Production, Università Cattolica del Sacro Cuore, Via Emilia Parmense, Piacenza, Italy

**Keywords:** Fruit expansive growth, functional–structural plant model (FSPM), grapevine, osmotic pressure, phloem hydraulic conductance, phloem sucrose concentration, transport, sink-driven carbon allocation, turgor pressure, xylem water potential

## Abstract

The growth of fleshy fruits is still poorly understood as a result of the complex integration of water and solute fluxes, cell structural properties, and the regulation of whole plant source–sink relationships. To unravel the contribution of these processes to berry growth, a biophysical grape (*Vitis vinifera* L.) berry growth module was developed and integrated with a whole-plant functional–structural model, and was calibrated on two varieties, Cabernet Sauvignon and Sangiovese. The model captured well the variations in growth and sugar accumulation caused by environmental conditions, changes in leaf-to-fruit ratio, plant water status, and varietal differences, with obvious future application in predicting yield and maturity under a variety of production contexts and regional climates. Our analyses illustrated that grapevines strive to maintain proper ripening by partially compensating for a reduced source–sink ratio, and that under drought an enhanced berry sucrose uptake capacity can reverse berry shrinkage. Sensitivity analysis highlighted the importance of phloem hydraulic conductance, sugar uptake, and surface transpiration on growth, while suggesting that cell wall extensibility and the turgor threshold for cell expansion had minor effects. This study demonstrates that this integrated model is a useful tool in understanding the integration and relative importance of different processes in driving fleshy fruit growth.

## Teaser

This study developed and used an advanced whole-plant grapevine model to unravel factors affecting water and carbon fluxes during fleshy fruit growth.

## Introduction

The growth of fleshy fruits largely depends on the balance of water influx and efflux (Lang and Thorpe, 1989; Lang, 1990). The flux of water into a fruit results from a tight co-ordination between vascular (xylem and phloem) transport and fruit cell expansion. The former is regulated by vascular conductivity and the water potential gradient between plant and fruit, and the latter by cell wall properties and the turgor of fruit cells (Lockhart, 1965; Matthews and Shackel, 2005). In fleshy fruits such as grape, which accumulate high concentrations of soluble sugars, carbon fluxes may also influence water flux by altering water potential gradients between the plant and fruit through changes in fruit osmotic potential (Coombe, 1992; Wada *et al.*, 2008, 2009; Keller and Shrestha, 2014; Zhang and Keller, 2017). Therefore, it is essential to investigate the regulation and co-ordination of water and carbon fluxes during expansive growth as they determine fruit yield and their ratio largely determines fruit composition, such as sugar concentration (Guichard *et al.*, 2001; Nardozza *et al.*, 2017; Kawasaki and Higashide, 2018).

The growth of a grape berry typically displays a double sigmoidal growth curve in which two phases of rapid growth, stages I and III, are separated by a lag phase, stage II (Coombe, 1992). The onset of ripening is referred to as véraison, and has been associated with the transition from stage II to stage III. At véraison, the resumption of rapid berry growth is accompanied by turgor loss, softening, sugar accumulation, organic acid degradation, cell wall loosening, xylem hydraulic changes, and colour accumulation in red cultivars (Coombe, 1992; Nunan *et al.*, 1998; Huang and Huang, 2001; Tyerman *et al.*, 2004; Castellarin *et al.*, 2016). At the same time, the main water transport pathway changes from xylem to phloem (Lang and Thorpe, 1989; Greenspan *et al.*, 1996; Keller *et al.*, 2015), and sugar transport shifts from the symplastic to apoplastic pathway (Zhang *et al.*, 2006). Similar changes are observed in other fleshy fruits (Morandi *et al.*, 2010; Clearwater *et al.*, 2012; Gould *et al.*, 2013; Brüggenwirth *et al.*, 2016).

The complex changes in berry physiology that occur at véraison make it difficult to differentiate the importance of each process in controlling the resumption of growth. The rapid growth of post-véraison berries occurs under an extremely low and relatively stable turgor of ~0.01–0.05 MPa (Thomas *et al.*, 2006, 2008; Matthews *et al.*, 2009; Castellarin *et al.*, 2016), and there is no correlation between growth rate and turgor of fruit cells (Matthews *et al.*, 1987). Therefore, it was postulated that post-véraison growth might be controlled by cell wall extensibility and/or a changing turgor threshold for cell expansion (Matthews *et al.*, 1987, 2009; Huang and Huang, 2001; Castellarin *et al.*, 2016). Cell wall composition and cell wall-modifying enzymes are indeed altered around véraison (Nunan *et al.*, 1998; Castellarin *et al.*, 2016), particularly with up-regulation of several genes encoding expansins, which promote cell wall loosening and cell wall disassembly (Schlosser *et al.*, 2008; Wong *et al.*, 2016). However, an alternative hypothesis considers the rapid sugar accumulation after véraison as the main driver of berry water influx, by increasing fruit osmotic potential and the water potential gradient between the plant and fruit, thus driving water influx (Coombe, 1960). The transcription of genes encoding sugar transporters is enhanced at véraison (Hayes *et al.*, 2007), as are those of some key sugar metabolism enzymes (Zhang *et al.*, 2006; Deluc *et al.*, 2007). Keller *et al.* (2015) reported that a sink-driven rise in sugar influx can counterbalance and even reverse berry contraction induced by water stress, highlighting the importance of sugar influx in regulating water flux.

Another aspect that could affect berry water influx is the vascular hydraulic conductance. Xylem hydraulic conductance of the pedicel shows a temporal increase around véraison and then gradually decreases until maturity in cvs Chardonnay and Grenache (Choat *et al.*, 2009; Scharwies and Tyerman, 2017). In Shiraz, xylem hydraulic conductance continuously decreased by >10-fold from young to mature berries (Tyerman et al., 2004). Despite those changes in xylem hydraulic conductance, berry water is mainly transported via phloem after véraison (Lang and Thorpe, 1989; Greenspan *et al.*, 1996; Ollat *et al.*, 2002; Keller *et al.*, 2015). The contribution of xylem hydraulic conductance to post-véraison berry growth, particularly as it varies between cultivars, remains open to question.

Water gained by berries through the vascular system can be lost by fruit transpiration, thereby modifying the driving force for water influx. The extent to which fruit transpiration determines water influx appears to vary with fruit developmental stage and environmental conditions. Grape berry transpiration decreases as the fruit develops, coinciding with a decrease in skin water conductance to water vapour of a grape berry, a key parameter of fruit transpiration (Zhang and Keller, 2015).

Furthermore, fruit growth is strongly impacted by the water and carbohydrate status of the parent plant, which are very difficult to assess experimentally (Lechaudel *et al.*, 2005; Lescourret *et al.*, 2011; De Swaef *et al.*, 2014; Hanssens *et al.*, 2015). A promising approach for analysing this integrated plant–fruit system is the use of process-based models such as functional–structural plant models, which represent a fruit tree virtually and allow the study of fruit growth behaviour *in silico* (Baldazzi *et al.*, 2013). The objectives of the present study were: to develop an integrative functional–structural plant model that can simultaneously simulate berry growth and whole-plant carbon and water status, and to use this model to unravel the key processes or parameters regulating berry growth, namely hydraulic conductance, sugar uptake, cell wall extensibility, berry surface transpiration, and plant water and carbon status. For simplicity, the current study focuses on post-véraison berry growth with a static plant architecture. Plant architecture here refers to the three-dimensional organization of the above-ground body such as the size and position of the shoots on a cordon and leaves on a shoot.

## Materials and methods

### Model overview

**Fig. 1.**
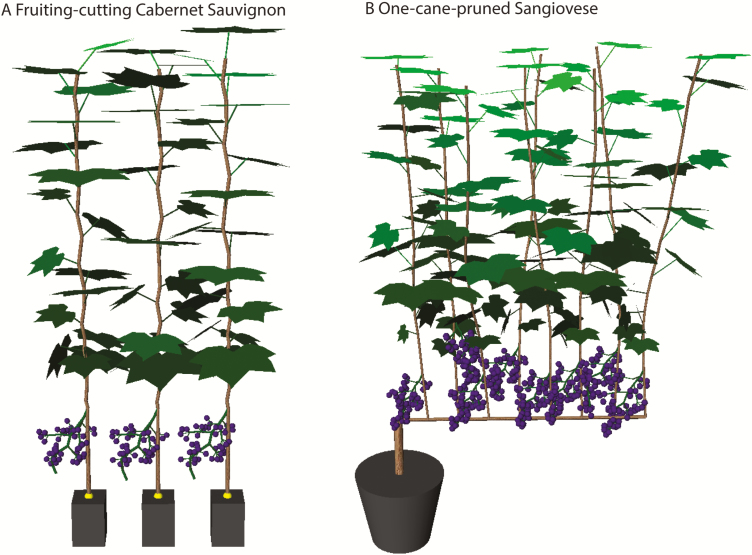
Illustration of the architecture of a fruiting-cutting Cabernet Sauvignon plant (A) and of a one-cane-pruned Sangiovese plant (B) in the model of GrapevineXL. The colour gradient across leaves represents the proportion of absorbed photosynthetically active radiation, which changes from black to light green as the proportion of absorbed photosynthetically active radiation increases. Photos for the experimental plant and condition are shown in Supplementary Fig. S1. The leaf area per plant for fruiting-cutting Cabernet Sauvignon was 0.104 m^2^ for 12 leaves per cluster and 0.025 m^2^ for three leaves per cluster. The leaf area per plant for one-cane-pruned Sangiovese was 1.02 m^2^ for 12 leaves per shoot, and 0.31 m^2^ for three leaves per shoot.

The current functional–structural grapevine model (GrapevineXL, Fig. 1) contains five main modules: (i) canopy architecture; (ii) leaf gas exchange; (iii) water transport; (iv) carbon allocation; and (v) berry growth. Detailed descriptions of the calculation and coupling of leaf gas exchange and water transport were provided in Zhu *et al.* (2018).

A sink-driven carbon allocation module was added to calculate the phloem sucrose concentration, which is an input variable for the berry growth module. The carbon allocation module calculates the phloem sucrose concentration based on the assumption that carbon loading is equal to carbon unloading at the whole-plant scale on an hourly basis (Baldazzi *et al.*, 2013). Xylem water potential and phloem sucrose concentration were assumed to be uniform throughout the plant, and were subsequently utilized by the berry growth module to simulate water and carbon uptake.

**Fig. 2.**
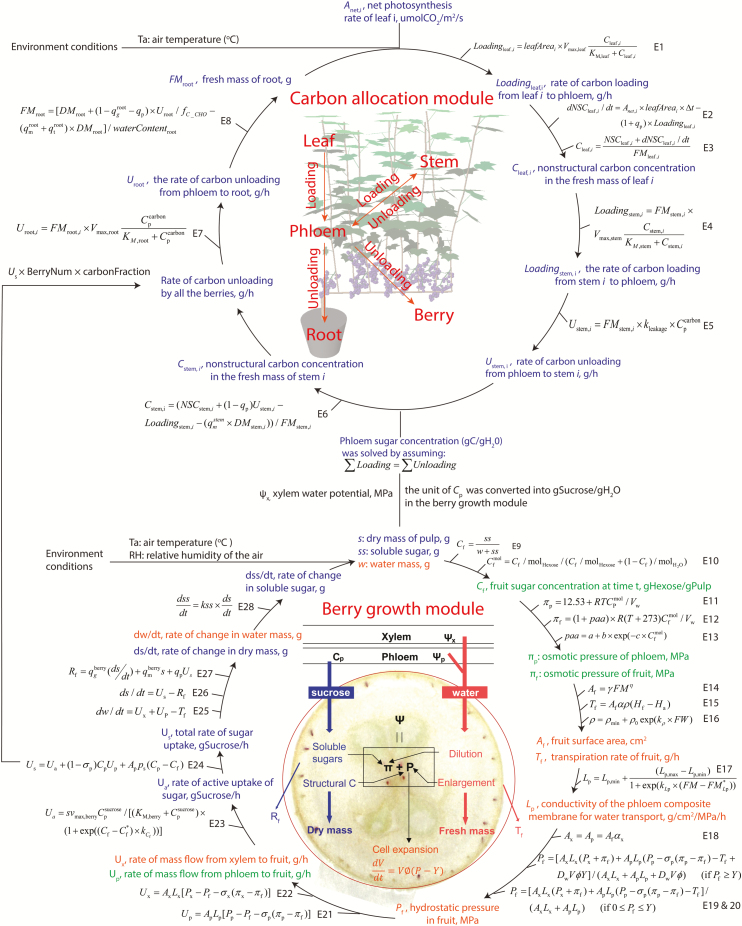
Schematic representation of the coupling of carbon allocation module and berry growth module in the model of GrapevineXL. The sink-driven carbon allocation module calculates the phloem sucrose concentration based on the balance between carbon loading from the leaf (E1) and stem (internode, cordon, and trunk, E4) and carbon unloading by berries (E24), roots (E7), and stem (E5). Subsequently, phloem sucrose concentration and xylem water potential, calculated by the water transport module (Zhu *et al.*, 2018), were utilized by the berry growth model. The berry growth module calculates water uptake from the phloem (or xylem) based on differences in hydrostatic and osmotic pressures between berry and phloem (or xylem, E21, and E22), and based on phloem (or xylem) water conductance (E17). Osmotic pressure was calculated from solute concentration (E11–E13). The phloem hydraulic conductance was assumed to decrease with increasing berry fresh weight (E17). Fruit hydrostatic pressure was calculated by solving Lockhart’s equation describing volume growth of the fruit and assuming that the volume change was equal to the total volume of water uptake from the xylem and phloem (E19 and E20). Water loss through berry transpiration was assumed to be proportional to the fruit surface area (E14) and surface conductance to water vapour (E16), and to be driven by the difference in relative humidity between the air-filled space within the fruit and the ambient atmosphere (E15). The sugar uptake was calculated based on the active transport mechanism (E23) and mass flow (E21 and E24). A constant fraction of increase in dry matter at each time step was converted into soluble sugar (E28), which enables the calculation of fruit sugar concentration (E9). Variables linked to carbon allocation processes are marked with blue, and variables linked with water transport are marked with orange. Variables linked with both processes are marked with green.

The berry growth module calculates water balance based on water uptake from xylem and phloem and water loss by fruit transpiration hourly. Meanwhile, berry dry mass accumulation was simulated through the balance between sucrose import from phloem and carbon depletion by respiration. Algorithms for the berry growth module and carbon allocation module are presented in Fig. 2 and in the following paragraphs.

### Berry growth module

The berry growth module was an adaptation of a fruit growth model originally developed for peach (Fishman and Génard, 1998; Dai *et al.*, 2008) and simulated the growth of an individual grape berry from the post-véraison developmental stage when cell division had ceased. In this module, berry growth was driven by two environmental variables (temperature and relative humidity), and two plant variables (xylem water potential and phloem sucrose concentration). The plant variables were calculated hourly by the whole-plant model. The berry growth module assumed that: (i) a grape berry can be considered as one compartment (a cell community with a constant number of growing cells) separated by a composite membrane from the parent vine and the outside environments; and (ii) the Lockhart equation originally applied to a single cell can describe the effect of hydrostatic pressure on the irreversible cell wall expansion in this average compartment (Lockhart, 1965; Fishman and Génard, 1998). A berry cluster was considered as a collection of berries, assuming all berries are identical. Thus total carbon or water uptake by a berry cluster equals the carbon or water uptake by a single berry multiplied by the number of berries.

Most of the post-véraison water gain is due to water import from the phloem (Lang and Thorpe, 1989). The water flow from phloem (or xylem) into the fruit was based on differences in hydrostatic and osmotic pressures between phloem (or xylem) and the berry, and phloem (or xylem) hydraulic conductance (Fig. 2). Osmotic pressure was calculated from the solute concentration. Fruit hydrostatic pressure (turgor) was calculated by solving Lockhart’s equation describing volume growth of the fruit, and assumed that the volume change was equal to the volume of water uptake from xylem and phloem minus berry transpiration. Water loss through berry transpiration was assumed to be proportional to the fruit surface area. The transport of sugars from the phloem to fruit mesocarp was described by: (i) mass flow, which is proportional to the solution flow at a given membrane reflection coefficient; and (ii) an active transport mechanism described by a modified Michaelis–Menten equation (Conde *et al.*, 2007). Passive diffusion, with the gradient of the sugar concentrations between phloem sap and berry flesh as a driving force, is negligible and was not considered (Fishman and Génard, 1998). Fruit photosynthesis was not considered because there is no fruit net carbon assimilation after fruit set (Lebon *et al.*, 2005).

Variables for the berry growth module are described in Fig. 2 and summarized in Supplementary Table S1, and parameter values are given in Table 1. Some modifications were made to the algorithms compared with the original model (Fishman and Génard, 1998), to take into account grape-specific properties:

**Table 1.** List of parameters in the berry growth module and carbon allocation module

Parameters	Definitions	Values		Unit	Sources^*a*^
		Cabernet Sauvignon	Sangiovese		
**Berry growth module**					
Berry surface area					
γ	Empirical coefficient	4.152	4.463	cm^2^ g^−1^	Experiment
η	Empirical coefficient	0.707	0.604	Dimensionless	Experiment
Berry surface transpiration					
ρ_min_	Minimum berry surface conductance to water vapour	55.4	25.8	cm h^−1^	Experiment
ρ_0_	Scaling factor	503	682	Dimensionless	Experiment
*k*_ρ_	Exponential decay rate	–4.97	–1.67	cm g^−1^ h^−1^	Experiment
*H*_f_	Relative humidity of air space in fruit	0.996		Dimensionless	Fishman and Genard (1998)
Phloem hydraulic conductance					
*L*_p,min_	Minimal phloem hydraulic conductance	3.5e-2		g cm^−2^ MPa^−1^ h^−1^	Exploration
*L*_p,max_	Maximal phloem hydraulic conductance	0.15	0.7	g cm^−2^ MPa^−1^ h^−1^	Calibration
FM*_Lp_	Fresh mass at the inflection point	0.95	1.33	g	Calibration
*k*_*L*p_	Proportional to the slope at inflection point of *L*p	9	7.4	g^−1^	Calibration
Composite membrane area					
α_x_	Coefficient for converting fruit surface area to membrane area	3.5e-3		Dimensionless	Calibration
Berry volume growth					
ϕ	Cell wall extensibility coefficient in Lockhart’s equation	0.1		MPa^−1^ h^−1^	Fishman and Génard (1998)
Y	Turgor pressure threshold for growth	0.05		MPa	Matthews *et al.* (2009); Castellarin *et al.* (2016)
Sugar uptake—mass flow					
σ_p_	Reflection coefficient for sugar for entering the composite membrane	0.9		Dimensionless	Fishman and Génard (1998)
Sugar uptake—active uptake					
*V*_max,berry_	Maximal rate of active sugar uptake per unit of dry mass	8e-3	2.8e-3	gSucrose (gDW)^−1^ h^−1^	Calibration
*K*_M,berry_	Michaelis constant for active transport	0.08		gSucrose gH_2_O^−1^	Milner *et al.* (1995); Fishman and Génard (1998)
C*_f_	Sugar concentration at the inflection point	0.13	0.15	gHexose gH_2_O^−1^	Calibration
*K*_*C*f_	Proportional to slope at the inflection point of *U*_a_	35		gH_2_O ghexose^−1^	Calibration
Sugar partition					
*k*_ss_	Fraction of increase in dry matter allocated into soluble sugar at each time step	0.9	1.0	Dimensionless	Experiment
*q*_m_^berry^	Maintenance respiration coefficient for berry	5.9e-5		gC gC^−1^ h^−1^	Dai *et al.* (2010)
*Q*_g_^berry^	Growth respiration coefficient for berry	0.02		gC gC^−1^	Dai *et al.* (2010)
Constants					
*V*_w_	Molal volume of water	18		cm^3^ mol^−1^	
*D*_w_	Water density	1		g cm^−3^	
R	Gas constant	8.3		cm^3^ MPa mol^−1^ K^−1^	
**Carbon allocation module**					
Carbon loading by leaf					
*V*_max,leaf_	Maximal rate of carbon loading per square meter of leaf per hour	1.0		gC m^−2^ h^−1^	Baldazzi *et al.* (2013)
*K*_M,leaf_	Michaelis constant for carbon loading by leaf	0.05		gNSC gFM^−1^	Exploration; Zufferey (2000); Quereix *et al.* (2001)
Carbon loading by internode, cordon, and trunk					
*V*_max,stem_	Maximal rate of carbon loading per gram of stem per hour	1.0e-4		gC gFM^−1^ h^−1^	Exploration; Grechi *et al.* (2007)
*K*_M,stem_	Michaelis constant for carbon loading by stem	0.05		gNSC gFM^−1^	Baldazzi *et al.* (2013)
Carbon unloading by internode, cordon, and trunk					
*k*_leakage_	Rate of carbon unloading per gram of stem per hour	3.5e-3		gC gFM^−1^ h^−1^	Exploration; Baldazzi *et al.* (2013); Rossouw *et al.* (2017)
Carbon unloading by root					
*V*_max,root_	Maximal rate of carbon unloading per gram of roots per hour	5e-4		gC gFM^−1^ h^−1^	Exploration; Barillot *et al.* (2016); Rossouw *et al.*, (2017)
*K*_M,root_	Michaelis constant for carbon unloading by roots	0.084		gNSC gH_2_O^−1^	Barillot *et al.* (2016)
Maintenance coefficient					
	Maintenance respiration coefficient	4e-5		gC gC^−1^ h^−1^	Cieslak *et al.* (2011)
	Maintenance respiration coefficient	2e-5		gC gC^−1^ h^−1^	Vivin *et al.* (2002)
	Maintenance respiration coefficient	2e-4		gC gC^−1^ h^−1^	Cieslak *et al.* (2011)
	Root turnover coefficient	2e-5		gC gC^−1^ h^−1^	Buwalda (1993)
Q10	Temperature ratio of maintenance respiration	2.03		Dimensionless	Thornley and Cannell (2000)
Growth coefficient					
	Growth respiration coefficient	0.2		gC gC^−1^	Vivin *et al.* (2003)
Carbon loading and unloading cost					
*q*_p_	Cost for either carbon loading to phloem or unloading from phloem	0.03		gC gC^−1^	Thornley and Cannell (2000)

^*a*^ Parameters were estimated in four complementary methods: (i) directly estimated from experimental data described above (experiment); (ii) directly taken from the literature; (iii) taken from the literature first but then adapted for grapevine based on the trends published in the literature or in our data collection (exploration); and (iv) taken from the literature first but then calibrated for our data through numerical optimization (calibration). The data sets of Dai *et al.* (2009) and Bobeica *et al.* (2015) were used for calibration.

(i) Berry surface conductance to water vapour deficit decreases with the increase in FW. This was in agreement with our measurements (Supplementary Fig. S2), and those reported by (Zhang and Keller, 2017).

$$\text{ρ}={\text{ρ}}_{\text{min}}+{\text{ρ}}_{0}\text{exp}(–k_{\text{ρ}}\times \text{FM})$$ (1)

where ρ was surface conductance to water vapour (cm h^−1^) and ρ_min_ was the minimum surface conductance. ρ_0_ and *k*_ρ_ were the fitted intermediate parameters.
(ii) The conductance of the phloem composite membrane for water transport was assumed to decrease with increasing FW. This assumption was based on the observation that the pedicel hydraulic conductance declined during ripening (Tyerman *et al.*, 2004; Knipfer *et al.*, 2015). We assumed that xylem hydraulic conductance was null after véraison, reflecting insignificant xylem inflow to the berry after véraison (Lang and Thorpe, 1989; Keller *et al.*, 2006) and that the current one compartment berry model cannot simulate xylem backflow because the water potential of the berry was more negative than the xylem potential.

$$L_{\text{p}}=L_{\text{p,min}}+\frac{\left(L_{\text{p,max}}-L_{\text{p,min}}\right)}{1+\exp[k_{L\text{p}}\times (\text{FM}-{\text{FM*}}_{L\text{p}})]}$$ (2)

where *L*_p_ was the phloem hydraulic conductance (g cm^−2^ MPa^−1^ h^−1^). *L*_p,min_ and *L*_p,max_ were the minimal and maximal phloem hydraulic conductance, respectively. FM*_Lp_ was the berry FW at the inflection point. *k*_*L*p_ was a scaling factor which was proportional to the slope at the inflection point of *L*_p_.
(iii) The rate of active sugar uptake per unit of dry mass was assumed to decrease with increasing berry sugar concentration. This assumption was based on the observation that the rate of sugar accumulation and invertase activity per gram of berry decreases at the later stage of berry ripening (Davies and Robinson, 1996), and berries that showed marked ripening state differences within a cluster at the véraison stage ultimately reached similar ripeness states toward maturity (Gouthu *et al.*, 2014). Furthermore, it has been shown that changes in the cellular concentrations of important signalling molecules such as sugars would affect the ripening process by influencing the expression of large networks of genes in yeast, Arabidopsis, and other species (Rolland *et al.*, 2006; Matsoukas *et al.*, 2013).

$$\begin{array}{l} U_{\text{a}}=sV_{\text{max,berry}}C_{\text{p}}^{\text{sucrose}}/\\ \langle \left\{K_{\text{M,berry}}+C_{\text{p}}^{\text{sucrose}}\}\times \{1+\exp[(C_{\text{f}}-C{*}_{\text{f}})\times k_{C_{\text{f}}}]\right\}\rangle \end{array}$$ (3)

where *U*_a_ was the active or facilitated sucrose transport per berry (gSucrose h^−1^), *s* was the dry mass of the pulp (g), and *V*_max,berry_ was the maximal rate of sucrose uptake per unit of pulp dry mass [gSucrose (gDW)^−1^ h^−1^]. *K*_M,berry_ was the Michaelis constant. $C_{\text{p}}^{\text{sucrose}}$ was the phloem sucrose concentration [gSucrose (gSolution)^−1^]. In the carbon allocation module, the phloem sucrose concentration was expressed as gram of carbon per gram of solution $C_{\text{p}}^{\text{carbon}}$ as we use carbon as the unit for calculating the carbon balance. *C*_f_ was the hexose concentration in the berry pulp [gHexose (gSolution)^−1^]. C*_f_ and *k*_*C*f_ described the inhibiting effects of fruit hexose concentration on sucrose uptake. The effect of seed number and micro-cracks on *V*_max,berry_ were not considered as we use the dynamics of mean berry weight and surface conductance to water vapour to calibrate the berry module.
(iv) A constant proportion of the increase in dry matter was allocated to soluble sugar. This is a simplified approach to represent the dynamics of soluble sugar, capturing the observed pattern that the fraction of soluble sugar in total dry mass increased over time from véraison to maturity (Dai *et al.*, 2009).

$$\frac{\text{d}ss}{\text{d}t}={\text{k}}_{\text{ss}}\times \frac{\text{d}s}{\text{d}t}$$ (4)

where *ss* was the soluble sugar in berry pulp (g), and *k*_ss_ was the fraction of increase in dry matter allocated to soluble sugar (mainly fructose and glucose) at each time step.

### Carbon allocation module

The carbon allocation module was adapted based on the model concepts and equations presented in Baldazzi *et al.* (2013). Briefly, carbohydrates stored in leaves and stem are loaded into the phloem at each time step (Fig. 2). Carbohydrates are then translocated to all sinks through the phloem network. Finally, carbohydrates are unloaded at the sink sites based on their carbon unloading capacities. Stem was just a simplified notation here for all internodes (current season shoot), cordons (2-year old shoot), and trunk (perennial woody part), although these objects were treated individually in the model. Phloem sucrose concentration was calculated based on the assumption that carbon loading from leaves and stem was equal to carbon unloading by stem, roots, and berries at each step (Fig. 2; Supplementary Protocol S1). Three types of respiration were considered (Table 1), namely phloem loading and unloading respiration (*q*_mobile_ for each process), maintenance respiration (*q*_m_), and growth respiration (*q*_g_). Growth respiration represents the carbon losses associated with the synthesis of new biomass. Growth respiration was calculated for the carbon unloaded to the root and berry but excluded for stem. We assume that the carbon unloaded to stem was mainly for temporary storage and can be reloaded into phloem in a short time, which was noted as a leakage-retrieval mechanism by van Bel (1996; Supplementary Protocol S1).

### Plant materials for model calibration and validation

Two sets of experiments were performed to calibrate and validate the model. The first set of experiments was done in a greenhouse with fruiting-cuttings of cv. Cabernet Sauvignon (Fig. 1; Supplementary Fig. S1) with two leaf-to-fruit ratios (Dai *et al.*, 2009; Bobeica *et al.*, 2015). Briefly, vines with one shoot and one cluster were pruned to either 12 or three main leaves per cluster (hereafter called 12LC and 3LC, respectively) at 1 week before véraison. Grape berries were harvested five times at 7 d intervals from véraison to maturity. DW, FW, hexose concentration (determined enzymatically Dai *et al.*, 2009), transpiration rate, and total osmolarity were measured. Berry transpiration rates were determined by weighing five detached berries with known diameter at hourly intervals during the daytime over 4–5 h under constant temperature (~20 °C) and vapour pressure deficit (~1 kPa). Total osmolarity was measured with a micro-osmometer (Roebling 13/13DR-Autocal, Berlin, Germany; Lechaudel *et al.*, 2007). An additional data set used for calibrating the response of photosynthesis and transpiration to soil water potential was described in Peccoux *et al.* (2017) and the calibration results were shown in Zhu *et al.* (2018). Parameters linked to canopy architecture, and the sizes and weights of internodes and leaves at different ranks were measured in a fruiting-cutting experiment in 2015 (Supplementary Protocol S2).

The second series of experiments was conducted using 4-year-old potted cv. Sangiovese vines with a 1 m long fruiting cane with eight or nine dormant buds. Detailed whole-canopy photosynthesis and transpiration, and the berry developmental profile were measured (Bobeica *et al.*, 2015). Vines were grafted on M_3_ rootstock and grown in 40 litre pots. Shoots were thinned to retain only one main shoot per node and one basal cluster. Two treatments with four replicates for each were applied: 1 week before véraison of 12LC or 3LC. Berries were sampled 14 times at 1 week intervals from 1 week before treatment to 8 weeks after treatment onset, and thereafter at 4 d intervals to better capture changes close to maturity. At harvest, all remaining berries of each vine were sampled, counted, and weighed. FW, DW, hexose concentration, berry transpiration, and total osmolarity were determined as described above.

Water was supplied automatically to avoid any water stress for all experiments. Moreover, hourly climate data, including temperature, relative humidity, radiation, and wind speed, were recorded in data-loggers throughout the experiments (Supplementary Fig. S3).

### Model inputs and initial conditions

The model uses hourly total radiation, air temperature, relative humidity, wind speed, CO_2_, and soil water content (or soil water potential) as the environmental input, and for plant status the dry mass of individual leaves, internodes, and roots as well as their structural and non-structural carbon fraction in the total carbon mass (Supplementary Table S2). For canopy architecture, the size of the blade, petiole, and internode, and the declination angle between the petiole and stem, and between the blade and petiole at different ranks are needed. To initialize the berry growth module, the model requires the number of berries per cluster, mean berry FW, DW, and hexose concentration at the beginning of simulation. Detailed model initiation methods for both fruiting-cutting Cabernet Sauvignon and one-cane-pruned Sangiovese are provided in Supplementary Protocol S2.

### Calibration of the berry growth module

The berry growth module was calibrated using the data sets of Dai *et al.* (2009) and Bobeica *et al.* (2015). The contributions of acids and other ions to total osmotic pressure at different soluble sugar concentrations were estimated using an exponential decay curve (E13 in Fig. 2; Supplementary Fig. S2; Supplementary Protocol S3). The Cabernet Sauvignon berry surface area was estimated using the recorded diameter of the berry by considering it as a sphere, and Sangiovese area was estimated using diameter and length from the proximal to distal position of the berry and assuming it to be ellipsoid. The relationships between berry surface area and FW (E14 in Fig. 2) were estimated by the non-linear least square method in the ‘stats’ library in R (R Development Core Team, 2017). Berry surface conductance to water vapour was calculated based on berry transpiration and surface area, and described as a function of berry FW through an exponential decay function (E16 in Fig. 2; Supplementary Fig. S2). *k*_*ss*_ in Equation 4 was estimated as the mean ratio between the increase of soluble sugar and the increase of dry mass between two successive sampling dates throughout the whole sampling period.

### Calibration of the carbon allocation module

Plant photosynthesis and transpiration for Sangiovese were first calibrated by the data set of Bobeica *et al.* (2015) (Supplementary Protocol S3). Parameters related to carbon export from leaf to phloem were estimated based on the diurnal dynamics of grapevine leaf non-structural carbon concentration published in Quereix *et al.* (2001) and Zufferey (2000). The ratio between *K*_m,berry_ and *K*_m,root_ (*K*_m,root_=2.5×*K*_m,berry_ in units of gC gH_2_O^−1^) was determined based on *K*_m_ values for grain and root in wheat (Barillot *et al.*, 2016). The value of *K*_m,berry_ was obtained from Milner *et al.* (1995) who measured the rate of sucrose transport of tomato tonoplast membrane at different sucrose concentrations. The remaining parameters were first taken from the literature (Table 1) and then explored by trial and error with fine refining, and optimizing afterwards (Supplementary Protocol S3).

Parameters linked with berry sugar and water uptake were calibrated separately for Cabernet Sauvignon and Sangiovese (Table 1), while most parameters linked with carbon allocation and water flux were kept the same for both systems. Final parameter calibration was done in the sequence of carbon unloading by the berry (*V*_max,berry_, *k*_*C*f_, and C*_f_) and water the uptake by berry (*L*_p,max_, FM*_Lp_, and *k*_*L*p_) through whole-plant model optimization. Parameters were calibrated at the whole-plant level by maximizing the sum of log-likelihood of the simulated model outputs given the observed berry DW and FW using the random walk Markov chain Monte Carlo (MCMC) method (Supplementary Fig. S4). Calibration was done based on the observed data of 12 leaves per cluster for both Cabernet Sauvignon and Sangiovese using the data set of Bobeica *et al.* (2015). The data of three leaves per cluster were reserved for validation. Validation was done by inputting the initial berry DW, FW, and hexose concentration at the start of simulation and then comparing the model output with the observed data. Berry sugar concentration was an emerging property of the model.

### Sensitivity analysis

To unravel the effects of different processes on berry FW and DW, a sensitivity analysis was done on all parameters within the berry growth module (Table 1). The default value of a parameter as noted in Table 1 was changed at 10% intervals from –50% to +50% excluding the default value, while all other parameters were kept at the default values. The FW or DW at the end of each simulation was used as the test variable. Simulations were run based on model settings for 12LC Cabernet Sauvignon and Sangiovese.

The sensitivity of the model to a given parameter was quantified by the normalized sensitivity coefficient, defined as the ratio between the percentage of changes in berry FW or DW $\Delta W/\bar{W}$ to the percentage of changes in parameter values $\Delta P/\bar{P}$ Equation 5).

$$\text{Sensitivity coefficient}=\frac{\Delta W/\bar{W}}{\Delta P/\bar{P}}$$ (5)

$\bar{W}$ is the final berry FW or DW under default parameter settings, while ΔW is the change in final berry FW or DW under the new parameter values in comparison with $\bar{W}$ Mean normalized sensitivity coefficients for the FW and DW were further calculated over the whole range of percentage changes for each parameter.

### Scenario simulations

Scenario 1: the effect of berry surface conductance on berry water balance was tested. Surface conductance to water vapour was set to zero, which was originally a function of berry FW.

Scenario 2: the effects of plant water status, *V*_max,berry_, and their interactions on berry FW and hexose accumulation were tested. Simulations were done for a 12 d period, mimicking the water stress–rewatering experiment described in fig. 2 of Keller *et al.* (2015). A drying and rewatering scenario was used with a period of water stress for the first 8 d (ψ_soil_ = –0.6 MPa) and then switched to a well-watered condition for the remaining 4 d (ψ_soil_= –0.05 MPa). Three different *V*_max,berry_ settings were tested to mimic the sharp increase of sugar unloading at véraison: (i) constant *V*_max,berry_ with the default value shown in Table 1${\bar{V}}_{\text{max,berry}}$ (ii) 0.1 ${\bar{V}}_{\text{max,berry}}$ for the first 4 d, and then a switch to ${\bar{V}}_{\text{max,berry}}$ for the remaining 8 d; and (iii) constant 0.1 ${\bar{V}}_{\text{max,berry}}$

Hourly climatic condition of a sunny day (7 August 2010) close to the véraison date in Bordeaux with a daily temperature range from 13 °C to 30 °C and total radiation up to 4000 µmol m^−2^ s^−1^ was used for the scenario simulation (Supplementary Fig. S5). The CO_2_ concentrations were maintained constant at 400 ppm, and ψ_soil_ for scenario 1 was maintained at –0.05 MPa. Simulations were done for 7 d. To make it easier to analyse the results, climatic conditions were assumed to be the same for each day.

All scenario simulations were done using the model settings for fruiting-cutting Cabernet Sauvignon, as the response of photosynthesis and transpiration of Cabernet Sauvignon to soil water potential has been calibrated in our previous study (Zhu *et al.*, 2018).

## Results

### Model calibration and validation

The functional–structural modelling approach enabled us successfully to simulate the hourly whole-plant photosynthesis and transpiration of the isolated potted Sangiovese vines under different leaf-to-fruit ratios based on environmental conditions (Supplementary Figs S6, S7). The model captured the increases in mean canopy photosynthesis and transpiration per unit of leaf area under three leaves per cluster (3LC) compared with 12 leaves per cluster (12LC; Supplementary Fig. S6), and illustrated that vines with 3LC allocated a greater proportion of carbon into berries than those with 12LC (73.1% versus 67.6% in Cabernet Sauvignon, 65.5% versus 52.2% in Sangiovese; Supplementary Figs S8, S9).

**Fig. 3.**
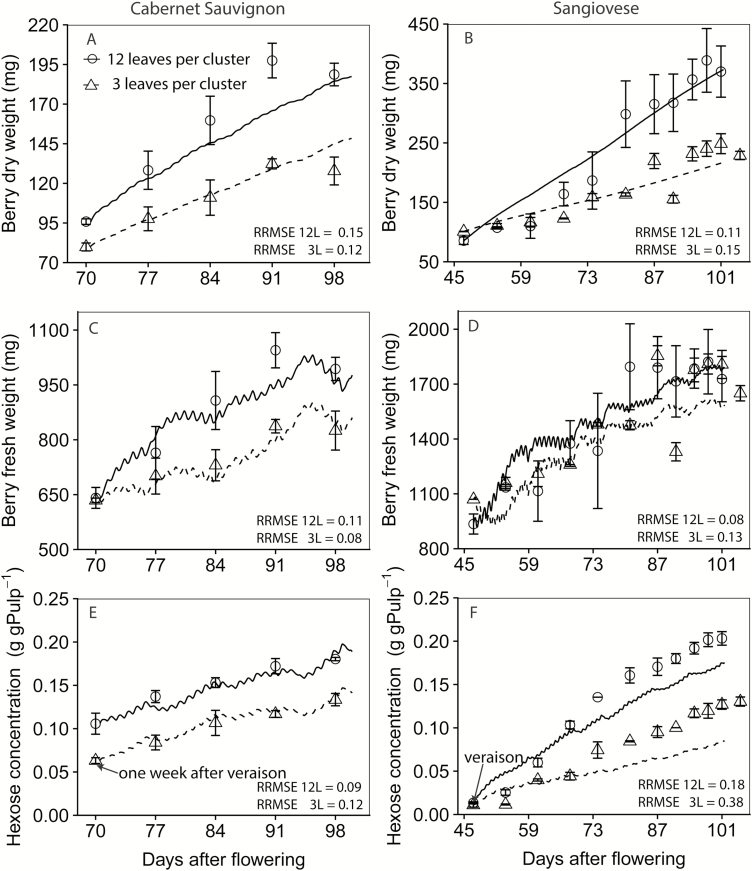
Model verification (12 leaves per cluster, solid lines) and validation (three leaves per cluster, dashed lines) of berry DW (A and B) and FW (C and D). Left panels are fruiting-cutting Cabernet Sauvignon, and right panels are one-cane-pruned Sangiovese. Circles and triangles are observed values, and lines are simulated values. The model was calibrated based on the dynamics of berry DW and FW under 12LC per cluster for using the data set of Bobeica *et al.* (2015) for both Cabernet Sauvignon and Sangiovese. The data set of 3LC per cluster was reserved for validation. The dynamics of berry hexose concentration was the emerging property of the model. RRMSE is the normalized root mean square error and represents the SD of the differences between predicted values and observed values divided by the overall mean of the observed values.

The model reproduced the dynamics of berry DW and FW under 12LC for both Cabernet Sauvignon and Sangiovese after calibration (Fig. 3), regardless of the contrasting starting conditions in berry weight and hexose concentration. It also predicted the negative effects of low leaf-to-fruit ratio (3LC) on DW, FW, and hexose concentration. The prediction for fruit hexose concentration was less robust than the prediction for DW and FW as we used a constant *k*_ss_ for estimating the dynamics of fruit hexose concentration without including specific enzymatic processes. Nevertheless, the predicted hexose concentration agreed well with the observed data for Cabernet Sauvignon (Fig. 3E), although it was lower than that observed for Sangiovese (Fig. 3F).

### Three major internal variables: xylem water potential, phloem sucrose concentration, and fruit turgor pressure

**Fig. 4.**
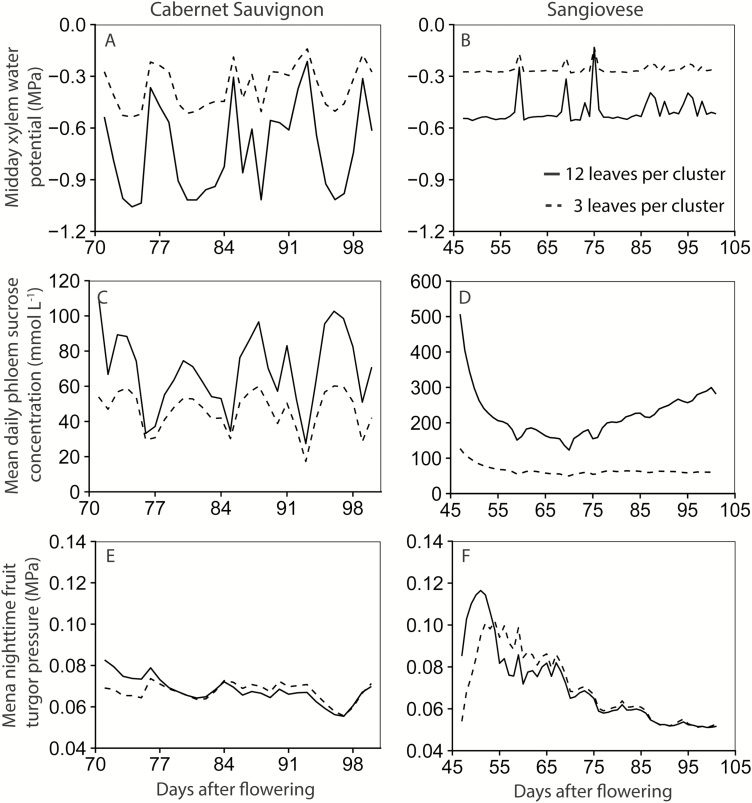
Mean mid-day xylem water potential (A and B), mean daily phloem sucrose concentration (C and D), and mean night-time turgor pressure (E and F). Left panels are fruiting-cutting Cabernet Sauvignon, and right panels are one-cane-pruned Sangiovese. The data sets of Bobeica *et al.* (2015) for both Cabernet Sauvignon and Sangiovese were used for the simulation. Solid lines represent the vines with 12 leaves per cluster, and dashed lines are vines with three leaves per cluster. The high phloem sucrose concentration at the start of the simulation could be because: (i) the input non-structural carbon concentration for leaf and stem was higher than the actual condition, thus the model requires some time to stabilize based on the current environmental condition; or (ii) berry has a lower sugar uptake capacity at the start of the simulation due to a lower dry matter.

The modelled mean mid-day xylem water potentials (considered to be in equilibrium with phloem water potentials) of the Cabernet Sauvignon were −0.73 MPa for 12LC and −0.36 MPa for 3LC (Fig. 4A). Similarly, 12LC showed a lower mean mid-day xylem water potential than 3LC (−0.50 MPa versus −0.26 MPa) in Sangiovese (Fig. 4B). Moreover phloem osmotic and turgor pressures fluctuated diurnally, with maximal and minimal values between 12.00 h and 16.00 h, respectively (Supplementary Fig. S10).

The modelled daily mean phloem sucrose concentration was 69.3 mM (mmol l^−1^) for 12LC and 46.2 mM for 3LC in Cabernet Sauvignon (Fig. 4C), while the average daily maximum $C_{\text{p}}^{\text{sucrose}}$ was 165.0 mM for 12LC and 80.1 mM for 3LC (Supplementary Fig. S11A). The daily mean $C_{\text{p}}^{\text{sucrose}}$ was 222 mM for 12LC and 64.9 mM for 3LC in Sangiovese (Fig. 4D), while the average daily maximum $C_{\text{p}}^{\text{sucrose}}$ was 258 mM for 12LC and 72.1 mM for 3LC (Supplementary Fig. S11B). The simulated daily mean $C_{\text{p}}^{\text{sucrose}}$ for 12LC Sangiovese was within the range, 125–1462 mM, reported by Jensen *et al.* (2013) in a meta-analysis on $C_{\text{p}}^{\text{sucrose}}$ with 41 plant species, although it was larger than the value reported for greenhouse grapevine (50 mM; Zhang *et al.*, 2006; Zhang and Keller, 2017). Furthermore, the model illustrated that $C_{\text{p}}^{\text{sucrose}}$ was greatly affected by the environmental conditions, such as radiation and soil water potential (Supplementary Fig. S12), and was positively related to the source:sink ratio. Increasing the source activity by raising the leaf number per cluster or by radiation, or decreasing the sink strength by reducing *V*_max, berry_, can cause an associated rise in $C_{\text{p}}^{\text{sucrose}}$ (Fig. 4; Supplementary Fig. S12).

The simulated night-time fruit turgor pressure decreased from véraison to maturity for both Cabernet Sauvignon and Sangiovese under both crop loads, ranging from 0.12 MPa to 0.05 MPa (Fig. 4E, F). This was consistent with measurements done by Matthews *et al.* (2009) in cvs Pinot Noir and Cabernet Sauvignon and by Castellarin *et al.* (2016) in cv Zinfandel, with a berry cell turgor of ~0.18 MPa at véraison and ~0.04 MPa at maturity.

### Berry water balance

**Fig. 5.**
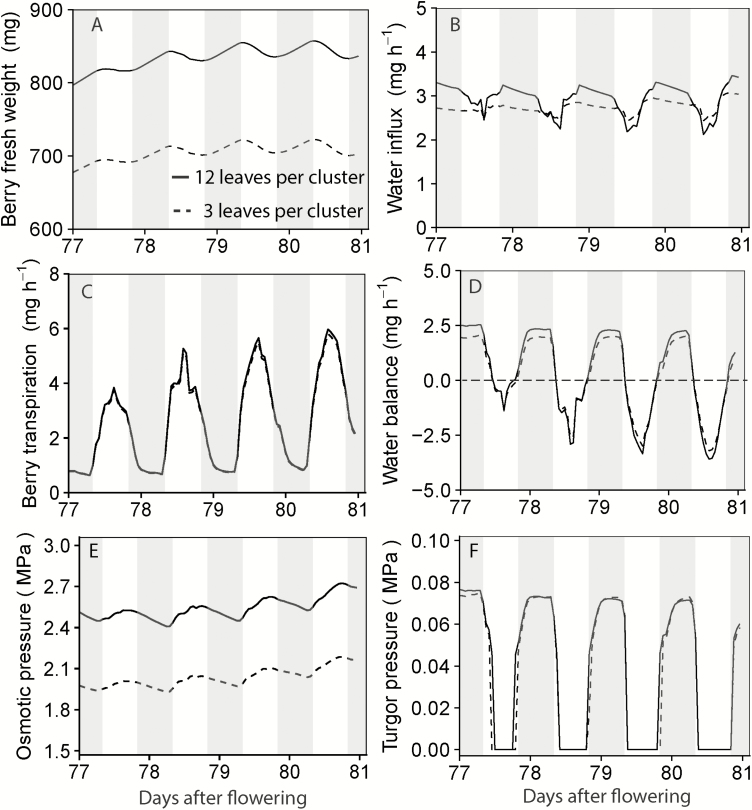
Simulations of diurnal dynamics of berry FW (A), water influx (B), surface transpiration (C), water balance (D), osmotic pressure (E), and turgor pressure (F) within a 4 d period (77–80 d after flowering) for Cabernet Sauvignon under a fruiting-cutting system. Solid lines were 12L per cluster, and dashed lines were 3L per cluster. Shaded areas indicated the night-time, 20.00 h to 05.00 h.

Berry FW fluctuated diurnally, with a predominantly negative water balance during the day, and a positive water balance at night (Fig. 5A, D). The negative water balance was largely caused by high berry transpiration during the daytime, which exceeded the water influx (Fig. 5B, C) under the experimental conditions for Cabernet Sauvignon. The lower water influx during the daytime compared with night-time (Fig. 5B) was due to a lower phloem water potential during the daytime (Fig. 4A). With respect to the negative water balance during the daytime, fruit turgor pressure was null during most of the day, but remained positive during the night-time (Fig. 5F).

### The sensitivity of berry growth to different processes

**Fig. 6.**
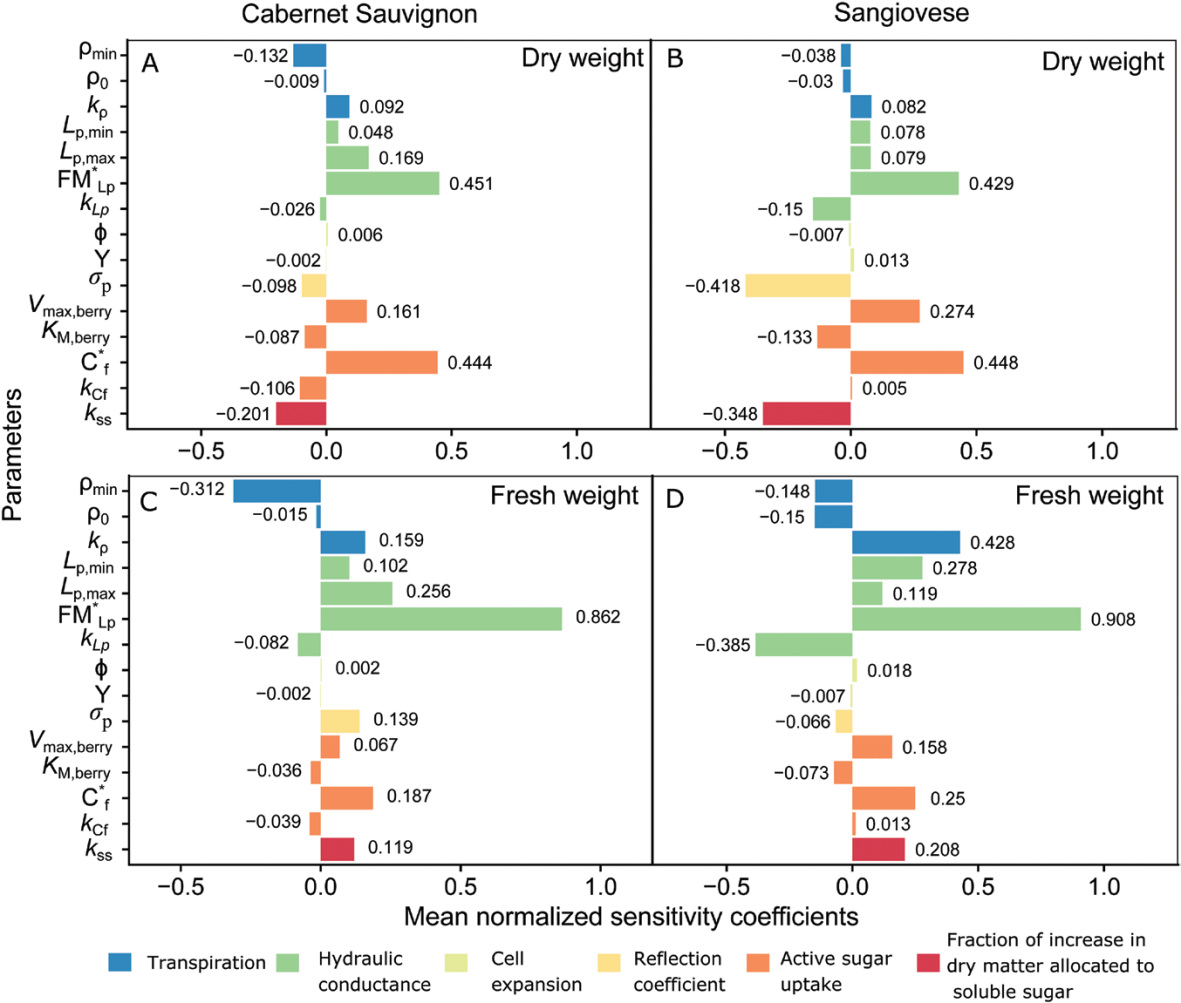
Mean normalized sensitivity coefficients (bars) calculated for the final berry DW (A and B) and FW (C and D) to variations in parameters within the berry growth module. The default value of a parameter as noted in Table 1 was changed at 10% intervals from –50% to +50%, excluding the default value, while all other parameters were kept at the default values during the sensitivity analysis. Left panels are Cabernet Sauvignon, and right panels are Sangiovese. Different coloured *V*_max,leaf_ represent different physiological processes.

Berry DW was most sensitive to parameters that control active sugar uptake (Fig. 6A, B), followed by parameters that control phloem hydraulic conductance, *k*_ss_, sugar uptake via mass flow, and berry surface transpiration. Relative sensitivities to different processes were similar between the two varieties. Among all the parameters, C*_f_ and FM*_Lp_ stood out, which were the inflection points for the logistic equations that calculate active sugar uptake (Equation 3) and phloem hydraulic conductance (Equation 2), respectively. The negative effect of *k*_ss_ on DW was due to the negative feedback of fruit sugar concentration on active sugar uptake that we include in Equation 3.

Concerning berry FW, the model was most sensitive to parameters that control phloem hydraulic conductance (Fig. 6C, D), followed by parameters that control berry surface transpiration, active sugar uptake, and *k*_ss_. FM*_Lp_ has the highest impact on berry FW across all the tested parameters. Neither DW nor FW was sensitive to cell wall extensibility and turgor threshold for cell expansion.

### The effect of berry surface transpiration on berry growth

**Fig. 7.**
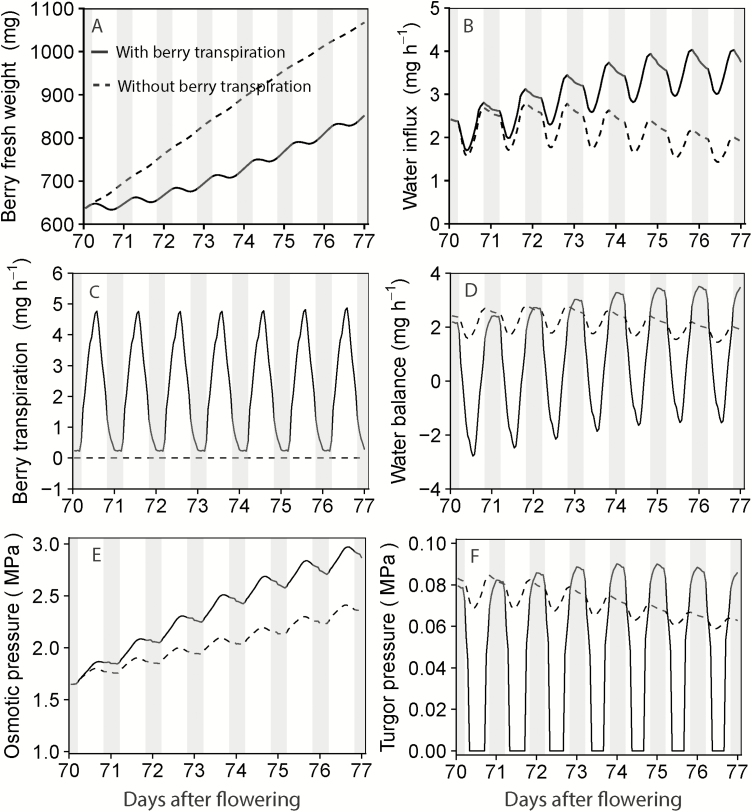
The dynamics of berry FW (A), water influx (B), surface transpiration (C), water balance (D), osmotic pressure (E), and turgor pressure (F) with surface transpiration (solid lines) and without surface transpiration (dashed lines). Simulation was run for 7 d based on the model set up for the fruiting-cutting Cabernet Sauvignon system. Climatic conditions are shown in Supplementary Fig. S5. Shaded areas indicated the night-time, 20.00 h to 05.00 h.

Preventing berry surface transpiration stimulated the increase of berry FW (Fig. 7A) largely due to a more positive water balance during the daytime (Fig. 7D). A rapid increase in berry FW resulted in a lower fruit osmotic pressure (Fig. 7E) and a higher fruit turgor pressure (Fig. 7F), which together gradually reduced the water influx (Fig. 7B). Furthermore, a steady increase in berry osmotic pressure under default conditions (solid line in Fig. 7E) resulted in a gradual increase in water influx (Fig. 7B).

The increase in berry surface area had little effect on berry transpiration as this was largely compensated by a reduction in berry surface conductance (Supplementary Fig. S2). As a result, the simulated berry transpiration remained stable over time (Fig. 7C).

### The effect of water deficit and berry sugar uptake capacity (**V**_max,berry_) on berry growth

**Fig. 8.**
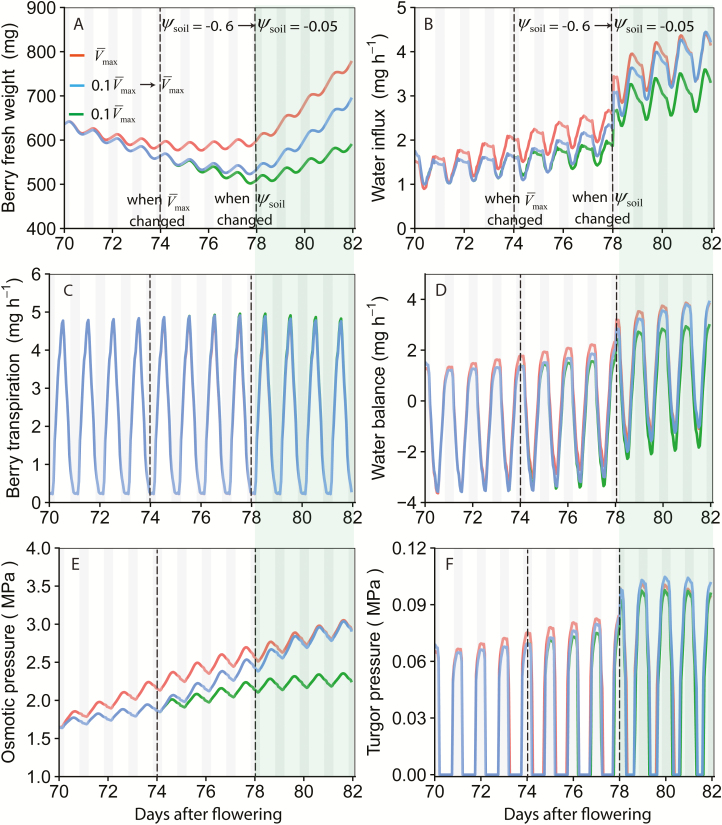
The dynamics of berry FW (A), water influx (B), berry surface transpiration (C), water balance (D), osmotic pressure (E), and turgor pressure (F) under varying sugar uptake capacity (*V*_max,berry_) with water stress for the first 8 d (70–77 d after flowering) and well watered for the remaining 4 d (78–81 d after flowering). Red lines were simulated with constant default *V*_max,berry_ (Table 1). Blue lines were simulated with 0.1*V*_max,berry_ for the first 4 d, and then switched to *V*_max,berry_ for the remaining 8 d. Green lines were simulated with 0.1*V*_max,berry_ throughout the whole period. Simulation was run based on the model set up for the fruiting-cutting Cabernet Sauvignon system. Climatic conditions are shown in Supplementary Fig. S5. Shaded areas indicated the night-time, 20.00 h to 05.00 h. The simulated dynamics of berry dry weight, hexose concentration, photosynthesis rate, transpiration rate, xylem water potential, and phloem sucrose concentration are shown in Supplementary Fig. S12.

Berry FW gradually decreased under water deficit (ψ_soil_= –0.6 MPa) for the first 4 d in all three *V*_max,berry_ scenarios (Fig. 8A). However, the scenario with constant default *V*_max,berry_ (red lines) stopped the decreases in FW from day 4 onwards (Fig. 8A) and started to result in a positive water balance (Fig. 8D). This was mainly caused by a faster increase in fruit DW, hexose concentration (Supplementary Fig. S12), and osmotic pressure (Fig. 8E) under a larger *V*_max,berry_. Increasing *V*_max,berry_ at day 5 (Fig. 8A blue lines) also slowed down the decline in berry FW and started to result in a gain in FW 4 d after the change.

Changing from the water-stressed condition (ψ_soil_= –0.6 MPa) to the well-watered condition (ψ_soil_= –0.05 MPa) instantly improved the plant water status and increased the rate of photosynthesis and $C_{\text{p}}^{\text{sucrose}}$ (Supplementary Fig. S12). This rapidly increased the water flux into the berry and induced more positive water balance and greater fruit turgor pressure (Fig. 8).

## Discussion

### Berry growth and its main drivers

This study developed a novel whole-plant grapevine model that simulates the effects of variations in environmental conditions (e.g. soil water potential, radiation, temperature, and vapour pressure), plant water status (e.g. xylem water potential, and leaf and fruit transpiration), and carbon status (e.g. source–sink ratio and phloem sucrose concentration) on post-véraison berry growth. The sensitivity analysis highlighted the importance of phloem hydraulic conductance, sugar uptake, and surface transpiration on berry growth (Fig. 6). A lower berry surface conductance to water vapour would reduce water losses by transpiration, although it was accompanied by a reduction in water influx into berries (Fig. 7). The reduction in water influx was mainly due to a decrease in plant-to-berry water potential gradient (Fig. 7). However, the weight gained by reduced transpiration was much larger than the loss due to decreased water influx (Fig. 7; 365 mg versus 155 mg over 7 d). This explains the increase in berry FW found in antitranspirant treatments (Rebucci *et al.*, 1997; Zhang and Keller, 2017).

A higher phloem hydraulic conductance would increase the water and sugar influx to the berry. Similarly, previous modelling work showed that phloem hydraulic conductance plays a major role in regulating tomato growth, and a tight positive correlation between pedicel phloem cross-sectional area and tomato fruit weight has been reported in various cultivars (Bussières, 2002). Interestingly, the dry mass of a grape bunch was positively correlated with the basal diameter of the bunch peduncle (Castelan-Estrada *et al.*, 2002), which may also suggest a relationship between berry growth and the abundance of phloem (consequently the phloem hydraulic conductance). Direct measurements of phloem hydraulic conductance in grape berry and pedicel may clarify these hypotheses and merit further exploration.

The model confirmed the hypothesis proposed by Coombe (1960) and Keller *et al.* (2015) that a rapid sugar accumulation after véraison is the main driver of berry water influx. Simulations showed that a high *V*_max, berry_ can help reverse the berry shrinkage under water deficit (Fig. 8), consistent with the observations of Keller *et al.* (2015). While the model confirmed the positive effects of *V*_max, berry_ on berry growth (Fig. 6), a paradox seems to exist: the *V*_max, berry_ of Cabernet Sauvignon was approximately three times that of Sangiovese (Table 1), while the fruit size of Cabernet Sauvignon is about half that of Sangiovese (Fig. 3). Meanwhile, we noticed that the phloem sucrose concentration in Cabernet Sauvignon is only 32% of that of Sangiovese (Fig. 4), because of the low radiation conditions in the greenhouse for Cabernet Sauvignon (Supplementary Fig. S3). These results led us to speculate on a potential biological compensation between *V*_max,berry_ and $C_{\text{p}}^{\text{sucrose}}$ in grape berry. To explore this speculation, we tested whether a similar final FW and DW could be reproduced for 12LC Cabernet Sauvignon with the *V*_max,berry_ and daily mean $C_{\text{p}}^{\text{sucrose}}$ of 12LC Sangiovese by running the berry growth module alone (carbon uptake did not affect $C_{\text{p}}^{\text{sucrose}}$ and vice versa. Simulation results confirmed this speculation. Thus the value of *V*_max,berry_ may not directly reflect the varietal differences as grape berry may be able to adjust *V*_max, berry_ under different plant carbon status to ensure the reproductive growth through either increases in enzymatic activity or the transcription of genes encoding sugar transporters. Further experimentation is needed.

However, one may question why the model can successfully reproduce the observed berry growth for 3LC treatment without implementing such a compensation in *V*_max, berry_. A further simulation was done by applying the larger *V*_max, berry_ of Cabernet Sauvignon to 3LC Sangiovese in the whole-plant model. The result showed that although there were 2-fold increases in *V*_max,berry_, the final DW only increased by 7.5%. Under strong source limitation, increases in *V*_max, berry_ would further deplete the limited carbon pool and reduce the $C_{\text{p}}^{\text{sucrose}}$ , resulting in small gains in carbon uptake. Previous studies showed that the percentage of carbon allocated to ripening berries increased under carbon limitation conditions, resulting in either no changes or decreases in final berry DW (Candolfi-Vasconcelos *et al.*, 1994; Di Lorenzo *et al.*, 2001; Rossouw *et al.*, 2017). A proportion of the carbon allocated to berries was remobilized from reserves in perennial and vegetative seasonal organs (Kliewer and Antcliff, 1970; Mansfield and Howell, 1981), especially the root system (Rossouw *et al.*, 2017).

The final berry FW of Sangiovese was approximately twice that of Cabernet Sauvignon (Fig. 3). Despite the potential difference in cell number, this may be caused by varietal differences in phloem hydraulic conductance and surface transpiration. Interestingly, Sangiovese has a higher *L*_p,max_ and FM*_Lp_, and a lower ρ_min_ than Cabernet Sauvignon (Table 1) which favour a larger berry, as illustrated with our sensitivity analysis and scenario simulation.

### Minor effects of cell wall extensibility and turgor threshold for cell expansion on post-véraison berry growth

The model indicated that cell wall extensibility and turgor threshold for cell expansion had minor effects on post-véraison berry growth (Fig. 6), although fully restricting cell wall extension would result in a rapid increase in berry turgor pressure and a reduction in water intake (Supplementary Fig. S13). This was in contrast to the sensitivity analysis done on the kiwifruit model (Hall *et al.*, 2013) where cell wall extensibility had a strong effect on cell expansion. The difference in the sensitivity of berry growth to cell wall extensibility probably arises from the differences in fruit sugar concentration and phloem hydraulic conductance. Grape berries have a much higher soluble sugar concentration (up to 25%) than kiwifruit (up to 8% at harvest; Hall *et al.*, 2013). A higher fruit sugar concentration means that osmotic potential would dominate fruit water potential. A larger osmotic potential can induce a larger water influx and can result in fruit growth even at low cell wall extensibility. Furthermore, the fitted maximum phloem hydraulic conductances for Cabernet Sauvignon and Sangiovese were 2 and 10 times, respectively, that of the constant phloem hydraulic conductance used in the kiwifruit model.

### Potential limitations of the model

While certain areas of knowledge are missing to represent the plant-fruit system accurately (e.g. phloem hydraulic conductance and phloem sucrose concentration in grapevine), this model provides insight into the integration and interactions of numerous processes during grape berry development. Two main potential limitations are listed below. 

First, carbon unloading processes from phloem to berry: Matthews *et al.* (2009) and Castellarin *et al.* (2016) found that a high fruit turgor pressure caused by restricting berry growth before véraison delayed the onset of véraison and sugar unloading. Similarly, applying gas pressure on the root of a fruiting vine before véraison increased berry FW, while delaying the onset of véraison and decreasing the sugar content per berry (Zhang and Keller, 2017). These findings indicated the potential existence of a turgor-dependent sugar unloading mechanism (Patrick, 1994), which is not captured by the current model. However, it is generally accepted that turgor-dependent unloading is more related to symplastic unloading where flow rate is a function of turgor pressure (Liesche and Patrick, 2017). In apoplastic sugar unloading mediated by energy-coupled carriers, as shown in the grape berry (Wang *et al.*, 2003; Zhang *et al.*, 2006), no clear linkage has been found between turgor pressure and the rate of sugar unloading (Pomper and Breen, 1996). The putative turgor-dependent sugar unloading behaviour observed in grape (Matthews *et al.*, 2009; Keller *et al.*, 2015; Castellarin *et al.*, 2016) might be related to the shift from symplastic to apoplastic unloading around véraison (Zhang *et al.*, 2006). However, it is still possible that some intermediate steps before apoplastic sugar unloading into the fruit would be affected by turgor pressure.

Secondly, xylem backflow: Zhang and Keller (2017) hypothesized that both berry transpiration and xylem backflow would serve as water discharge pathways to facilitate phloem unloading and sugar accumulation during grape ripening. Xylem backflow means that there is excessive phloem water influx, which could be reflected by the current model when the simulated ratios between sugar and water uptake from phloem were greater than the phloem sucrose concentration. The simulations indicated that xylem backflow or lateral water flow from phloem to xylem (Hall and Minchin, 2013) would occur when the phloem sucrose concentration was low, especially for Cabernet Sauvignon. However, we cannot directly simulate xylem backflow because: (i) we treated the berry as a single fruit compartment with one composite membrane separating the berry and the parent plant, and assumed that the fruit was directly connected to the plant stem; and (ii) the fruit water potential was always low. To solve that problem, an apoplast compartment could be required. A recent published kiwifruit model has demonstrated its ability in simulating xylem backflow by including an apoplast component, although the authors only show moderate xylem backflow at mid-day (Hall *et al.*, 2017).

### Conclusion

A new whole-plant grapevine model was developed for assessing the contribution of different physiological processes to berry growth and the observed variations in growth caused by either exogenous or endogenous resource availability. The model showed that phloem hydraulic conductance, active sugar uptake, and berry transpiration have a major influence on post-véraison berry growth, and suggested that berries may be able to increase the maximum rate of sucrose uptake per unit of biomass under stress conditions. The ability of the model in testing the importance of different processes and environmental conditions on berry growth could assist breeders to define the ideal variety for certain environments. Furthermore, the model can easily be transferred into different grapevine training systems and help identify the potential yield under novel training systems and best management options: irrigation (amount and schedule), crop load, and plant architecture management.

## Supplementary data

Supplementary data are available at *JXB* online.

Table S1. List of variables in the berry growth module.

Table S2. List of variable values for initializing the model.

Protocol S1. Carbon allocation module.

Protocol S2. Model set up and initialization.

Protocol S3. Calibration procedure for the berry growth module, whole-plant photosynthesis, and carbon allocation module.

Fig. S1. Illustration of the experimental condition.

Fig. S2. Correlation between berry surface conductance to water vapour and FW, and the contribution of other compounds to total osmotic pressure.

Fig. S3. Climate conditions during the experimental period.

Fig. S4. Evolution of *V*_max,berry_ and log-likelihood during one of the parameter optimizations.

Fig. S5. Diurnal climatic conditions used for the scenario simulations.

Fig. S6. Verification and validation of the diurnal dynamics of photosynthesis, transpiration, and water use efficiency.

Fig. S7. Observed versus simulated hourly photosynthesis, transpiration, and water use efficiency.

Fig. S8. Diurnal carbon loading by leaf and stem.

Fig. S9. Daily mean fraction of carbon unloading by berries, stem, and roots.

Fig. S10. Diurnal changes of phloem osmotic pressure, turgor pressure, and water potential.

Fig. S11. Maximum and minimum daily phloem sucrose concentration.

Fig. S12. The dynamics of berry DW, fruit hexose concentration, mean canopy photosynthesis rate, transpiration rate, xylem water potential, and phloem sucrose concentration with varying sugar uptake capacity under water stress and rewatering scenarios.

Fig. S13. The effects of no cell wall extensibility on berry growth.

Supplementary Material
