# Supplementary material for: Modelling grape growth in relation to whole-plant carbon and water fluxes
Source: J Exp Bot. 2018 Oct 23;70(9):2505–21. doi: 10.1093/jxb/ery367 (PMC6487596; doi:10.1093/jxb/ery367)
Supplement: Supplementary Material [file ery367_suppl_supplementary_material.pdf]

Supplementary Data

Modelling grape growth in relation to whole-plant carbon and water fluxes

Junqi Zhu[1,2], Michel Génard[3], Stefano Poni[4], Gregory A. Gambetta[1], Philippe Vivin[1], Gilles Vercambre[3], Michael C. T. Trought[2], Nathalie Ollat[1], Serge Delrot[1], Zhanwu Dai[1*]

1. EGFV, Bordeaux Sciences Agro, INRA, Université de Bordeaux, ISVV, 33140 Villenave d'Ornon, France

2. The New Zealand Institute for Plant and Food Research Limited (PFR) Marlborough, Blenheim 7240, New Zealand

3. INRA, UR 1115 Plantes et Systèmes de Culture Horticoles, Avignon, France

4. Istituto di Frutti-Viticoltura, Università Cattolica del Sacro Cuore, Via Emilia Parmense 84, 29122 Piacenza, Italy

Author for correspondance: Zhanwu Dai
Email: zhanwu.dai@inra.fr
Tel: +33-(0)5 5757 5922

Fax: +33-(0)5 5757 5003

Table S1. List of variables in the berry growth module

Variables	Description	Unit
A_f	Fruit surface area	cm^2
C_f	Concentration of sugar in fruit pulp (mainly hexose in this case)	$g\ g^{-1}$
$C_p^{sucrose}$	Phloem sucrose concentration	g Sucrose $g\ H_2O^{-1}$
C_p^{carbon}	Phloem carbon concentration	g Carbon $g\ H_2O^{-1}$
DM	Total dry mass of the fruit, include pulp and seed	g
FM	Total fresh mass of the fruit	g
f_s	Fraction of soluble sugar in the dry mass of the pulp	dimen-sionless
NSC	Non-structural carbon	g
P_f	Hydrostatic pressure in fruit	MPa
P_p	Hydrostatic pressure in phloem	MPa
P_x	Hydrostatic pressure in xylem	MPa
R_f	Fruit respiration rate	$g\ h^{-1}$
s	Dry mass of the pulp per fruit	g
T_f	Fruit transpiration rate	$g\ h^{-1}$
U_a	Rate of active uptake of sugar	$g\ h^{-1}$
U_p	Rate of mass flow from phloem to fruit	$g\ h^{-1}$
U_x	Rate of mass flow from xylem to fruit	$g\ h^{-1}$
U_s	Total rate of sugar uptake	$g\ h^{-1}$
w	Amount of water mass in the pulp per fruit	g
ρ	The conductance of fruit surface to water vapour	$cm\ h^{-1}$
π_f	Osmotic pressure in fruit	MPa
π_p	Osmotic pressure in phloem	MPa
π_x	Osmotic pressure in xylem	MPa
ψ_x	Water potential in xylem	MPa
ψ_f	Water potential in fruit	MPa

Table S2. list of variable values to initialize the model

Input variables	Fruiting-cutting Cabernet Sauvignon	one-cane-pruned Sangiovese	Description
Latitude (degree)	44.8	45	the latitude of the simulation place
Simulation control			
nrRows	3	1	number of rows
nrPlants	5	4	number of plants in a row
rowDistance (meter)	0.15	1.10	distance between rows
plantDistance (meter)	0.15	1.10	distance between plants in a row
Plant morphology			
CORDON_NUM	1	1	number of cordons per vine
SHOOT_NUM	1	8	number of shoots per cordon
MAX_LEAF_NUMBER	15	15	number of leaf per plant
STARTING_LEAF_NUMBER	4	4	starting node position
lwRatio	1.4	1.4	ratio between leaf blade length and width
shapeCoeff	2	2	leaf shape coefficient (0 rectangular,high value pinched)
phyllotaxis (degree)	137.5	137.5	angle between consecutive leaves alonga stem
Leaf optical properties			
reflectancePAR	0.1	0.1	reflectance of PAR by leaves and stem
transmittancePAR	0.05	0.05	transmittance of PAR by leaves
Initial condition			
SLA (cm^2 g^{-1})	200	45.4	cm2 g specific leaf area,for calculating biomass
BIOMASS_INTERNODE (mg)	2510	by_density_length	biomass at veraison
BIOMASS_ROOT (mg)	1866.25	106666.8	biomass at veraison
BIOMASS_WOOD (mg)	12286.875	by_density_length	biomass at veraison
BIOMASS_CORDON (mg)	3071.71875	by_density_length	this is for one cordon
BERRY_FRESH_WEIGHT (mg)	691	985	mean berry Fresh weight includeSeed at verasion
BERRY_DRY_WEIGHT (mg)	120	110	mean berry dry weight at verasion include seed
CONENTRATION_SOLUBLE_SUGAR	0.105	0.014	gSolubleSugar gH20 at veraison
berryNum	45	92	number of berries per bunch
SEED_FRESH_MASS (mg)	50	50	The fresh weight of seed at veraison

leafNContent_input (g m^{-2})	1.5	2.1	leaf nitrogen content per square meter
Calculation of wood shoot biomass based on density			
WOOD_DENSITY (mg/cm3)		400	density of the wood
WOOD_DIAMETER (meter)	0.01	0.03	Diameter of the wood part
WOOD_LENGTH (meter)	0.2	0.35	Length of the wood part
CORDON_DENSITY (mg/cm3)		400	the density of shoot may change with age
CORDON_DIAMETER (meter)		0.012	Diameter of the cordon part
CORDON_LENGTH (meter)		1	Length of the cordon part
SHOOT_DENSITY (mg/cm3)		300	the density of shoot may change with age
SHOOT_DIAMETER (meter)	0.008	0.008	Diameter of the wood part
SHOOT_LENGTH (meter)	1	1	Length of the wood part
Water concentration of each organ			
WATER_CONTENT_LEAF	0.76	0.76	data at veraison
WATER_CONTENT_ROOT	0.9	0.8	data at veraison
WATER_CONTENT_INTERNODE	0.8	0.8	data at veraison
WATER_CONTENT_WOOD	0.56	0.56	data at veraison
WATER_CONTENT_BERRY	0.82	0.82	data at veraison
Carbon content in whole biomass			
C_CONTENT_LEAF	0.439	0.439	data at veraison
C_CONTENT_INTERNODE	0.442	0.442	data at veraison
C_CONTENT_ROOT	0.420	0.420	data at veraison
C_CONTENT_BERRY	0.433	0.433	data at veraison
C_CONTENT_WOOD	0.464	0.464	data at veraison
Structural carbon in total carbon			
STRUCTURE_C_LEAF	0.863	0.863	data at veraison
STRUCTURE_C_INTERNODE	0.898	0.898	data at veraison
STRUCTURE_C_ROOT	0.904	0.904	data at veraison
STRUCTURE_C_BERRY	0.082	0.082	data at veraison
STRUCTURE_C_WOOD	0.903	0.903	data at veraison
Sucrose starch carbon in total carbon			
SUCROSE_C_LEAF	0.136	0.136	data at veraison
SUCROSE_C_INTERNODE	0.102	0.102	data at veraison
SUCROSE_C_ROOT	0.095	0.095	data at veraison

SUCROSE_C_BERRY	0.917	0.917	data at veraison
SUCROSE_C_WOOD	0.0967	0.097	data at veraison
External input files			
leafDeclinationAngle_file	Exp2015_Zhu_Bordeaux_leaf.declination.angle.csv		the declination angle between petiole and stem, and between blade and petiole at different ranks
organSize_file	Exp2012_Dai_organSize	Exp2013_Bobeica_organSize	organ size of leaf, internode, petio along ranks
climate_file	Exp2012_Dai_climate	Exp2013_Bobeica_climate	Climate file
berryWeight_file	Exp2012_Dai_berryProfile	Exp2013_Bobeica_berryProfile	The dynamics of berry weight

Supplementary Protocol S1: detailed description of the carbon allocation module

A diagram of the carbon allocation module was presented in Fig. 2 in the main text, and parameter values were listed in the table 1 of the main text.

Carbon loading from leaf to phloem was assumed to be an active loading process, and was modelled as Michaelis-Menten kinetics (E1 to E3 in Fig. 2). The rate of carbon loading ($Loading_{\text{leaf,i}}$) was determined by the area of each individual leaf ($A_{\text{leaf,i}}$), maximum rate of carbon loading per unit of leaf area ($V_{\text{max,leaf}}$), the non-structural carbon concentration per unit of leaf fresh mass ($C_{\text{leaf,i}}$), and a Michaelis constant ($K_{\text{M,leaf}}$). Similar to carbon loading from leaf to phloem, a Michaelis-Menten function was also used for carbon loading from stem to phloem (E4 in Fig. 2) based on the description in Patrick *et al.* (2001).

Loss of assimilates from the phloem to the stem parenchyma may occur through apo- or symplasmic pathways (Patrick and Offler, 1996; Van Bel, 1996). This process was noted as leakage in Patrick *et al.* (2001). It was modelled as a multiplicative function (E5 in Fig. 2) of phloem carbohydrate concentration ($C_{\text{p}}^{\text{carbon}}$), fresh mass of stem_i (FM_{i}), and a constant unloading rate per unit of fresh mass per hour (k_{leakage}). Leakage and reloading of carbohydrates occur simultaneously along the phloem pathway.

Carbon unloading by root was simulated as an active unloading process (E7 in Fig. 2), following Barillot *et al.* (2016). Carbon loading from root to phloem was ignored as root was a sink rather than a source at post-véraison stage in grapevine. The rate of carbon unloading by root (U_{root}, E7 in Fig. 2) depended on the fresh mass of the root (FM_{root}), maximum carbon unloading rate per unit of fresh mass ($V_{\text{max,root}}$), phloem carbon concentration ($C_{\text{p}}^{\text{carbon}}$), and a Michaelis constant ($K_{\text{M,root}}$).

Three types of respiration were considered in this study (Table 1), named growth respiration (q_{g}), phloem loading respiration and unloading respiration (q_{mobile} for each process) and maintenance respiration (q_{m}). The effect of temperature on the rate of maintenance respiration was included through a Q10 concept (De Vries and Van Laar, 1982). For perennial fruit crops, it was speculated that fine roots would turn over within 1 year while the growth of structural roots were negligible (Buwalda, 1993; Janssens *et al.*, 2002; Cieslak *et al.*, 2011). According to Buwalda (1993) fine roots were less than 20% of the total root biomass, thus the loss of root biomass through root death ($q_{\text{d}}^{\text{root}}$) was estimated as 2e-5 gC gC^{-1} h^{-1} (≅ 0.2/365/24). Growth respiration for stem was not considered in this study as we focus on the post véraison stage with static architecture. We only consider the process of carbon unloading from phloem

to stem, and carbon loading from stem to phloem. Maintenance coefficient for leaves was excluded as maintenance respiration was already included in the calculation of dark respiration in the extended-FvCB module.

Supplementary Protocol S2: Model set up and initialization

A list of model input variables were shown in Table S2.

The size of leaf, internode and petiole, the declination angle between petiole and stem, and between blade and petiole at different ranks of Cabernet Sauvignon under fruiting-cutting conditions were determined in the experiment of 2015. Leaves were mostly opposite to each other for grapevine, so phyllotaxis was set to 180 degree. However, the horizontal angle between petiole and blade could change based on the environmental condition, such as the direction of the light. We did the leaf azimuth measurement on fruiting-cutting Cabernet Sauvignon in the greenhouse, but did not find any patterns along the different ranks. A random number between -10 to 10 was given for the leaf azimuth.

The biomass of each component leaf, internode, trunk (wood cuttings) and root as well as water content at véraison stage were determined in 2015. The concentrations of non-structural carbon in each component were derived from the experiment described in Ollat and Gaudillere (1998) for fruiting-cutting Cabernet Sauvignon. The number of berries per bunch (45 berries per bunch at véraison), leaf mass, specific leaf area, fresh weight, dry weight and hexose concentration were determined in the experiment of 2012, and were input into the model as the initial condition. Leaf area was estimated using the relationship between specific leaf area (m^2 fresh area gDW^{-1}) and total leaf dry weight. The biomass of internode, trunk and root were assumed to be the same as the experiment of 2015. Two berries per bunch were removed every 7 days during simulation in accordance with the sampling procedure.

The architecture for the one-cane-pruned Sangiovese was set up in the model based on the mean trait value of four vines. A Sangiovese plant was configured to have eight shoots on a 1-m fruit-bearing cane. The size distributions of leaf and internode along ranks were assumed to be the same as fruiting-cutting Cabernet Sauvignon. The length of each leaf was multiplied by 1.4 to obtain the observed mean leaf area per vine (1.02 m^2 $vine^{-1}$ with 8 shoots for the treatment of 12 leaves per cluster, 0.31 m^2 $vine^{-1}$ with 8 shoots for 3 leaves per cluster). The length and diameter of the trunk, cordon and shoot were estimated in ImageJ based on images taken during the experiment. The number of berries per cluster was set as 92 for the treatment of 12 leaves per cluster, and set as 110 for the treatment of 3 leaves per cluster based on the experimental record. Leaf area was measured on the leaves that were removed the day of treatment and after harvest of all berries (Bobeica *et al.*, 2015). Leaf area was determined by measuring the surface of each blade with a leaf area meter (LI-3000A, LI-COR Biosciences, Lincoln, NE, USA). Specific leaf areas were determined and were the same between the 12-

leaf and 3-leaf treatments. Three berries per cluster were removed after each sampling date. Similarly in model simulation three berries per cluster were removed every 7 days.

The biomass of the leaf was calculated based on the observed leaf area and specific leaf area in Sangiovese (Bobeica *et al.*, 2015). The biomass of trunk, cordon and shoot were calculated based on length, diameter, and wood density (400 mg cm^3 for trunk and cordon, and 300 mg cm^3 for shoot)(Castelan-Estrada *et al.*, 2002). The biomass of the root was estimated by the relationship between shoot fresh weight and root fresh weight presented in Poni *et al.* (1992).

For fruiting-cuttings of Cabernet Sauvignon, fifteen plants (three rows with five plants per row) were described in a 3D scene based on the plant configuration in the greenhouse. The mean of the fifteen plants was used in the calculation and optimization. For one-cane-pruned Sangiovese, four plants in one row were described in a 3D scene. The mean of the four plants was used in the calculation and optimization.

Supplementary Protocol S3: calibration of the berry growth module, whole-plant photosynthesis and carbon allocation module

Calibration of the berry growth module

In the Lockhart cell growth equation, the threshold value of turgor pressure (Y) above which expansive growth occurs was fixed at 0.05 MPa (Table 1), based on direct turgor measurement on post-véraison grape berries (Thomas *et al.*, 2006; Thomas *et al.*, 2008; Matthews *et al.*, 2009; Castellarin *et al.*, 2016). As the coefficient describing the cell wall extensibility in grape has not been reported, the value from peach (0.1 $MPa^{-1}h^{-1}$) was used (Fishman and Génard, 1998). The reflection coefficient describing the impermeability of berry cell membranes was set at 0.9, reflecting the substantial membrane integrity maintained over normal berry development (Krasnow *et al.*, 2008). Parameters relating with fruit surface area, skin surface conductance to water vapour, the contribution of hexose to total osmolarity and sucrose allocation at each time step (k_{ss}) were estimated based on experimental measurements and their values were given in Table 1 in main text and Supplementary Figs. S2.

Whole plant canopy photosynthesis

The biochemical photosynthesis parameters for Sangiovese were assumed to be the same as Cabernet Sauvignon as shown in Zhu *et al.* (2018). The performance of the model in predicting the whole-canopy photosynthesis of Sangiovese with 12 leaves per cluster was optimized by adjusting leaf nitrogen content and the response of stomata conductance to vapour pressure deficit (VPD). Parameters were optimized by the Metropolis-Hastings algorithm with random walk Markov chain Monte Carlo (MCMC) method at the whole-plant level. The method was customized written within the whole-plant model with the basic java common math library. This algorithm accepts a new parameter set when the difference between the log-likelihood calculated based on the new parameter and previous log-likelihood is larger than the natural logarithm of a random value between 0 and 1. Parameters were optimized only based on the photosynthesis, transpiration and water use efficiency data between 10 o'clock to 16 o'clock and filtered by water use efficiency (unit: umol CO_2 $mmolH_2O^{-1}$) smaller than four and larger than two, which were the most reliable data section according to our experience with this gas exchange measurement. The optimization result was further verified with the all whole-canopy photosynthesis records of 12 leaves per cluster and of 3 leaves per cluster.

Calibration of the carbon allocation module

Parameters related with carbon allocation were first taken from literature (Table 1 main text) and then explored by try and error to ensure the simulated trends was agree with our general knowledge. Parameters linked with carbon unloading by stem and root ($k_{leakage}$ and $V_{max,root}$) were fixed after many rounds of parameter exploration which produced a general trends that there is around 10% increase in root biomass and 20% increase in stem biomass from véraison to harvest with berry dry mass is close to the observed values in the 12 leaves per cluster treatment in one-cane-pruned Sangiovese. This trend is shown by Rossouw *et al.* (2017) that root biomass increased by 5 to 15%, trunk biomass increased by about 13% and shoot biomass increased by about 42% from véraison to harvest in potted vine with full irrigation and a leaf to fruit ratio around 1.8 m^2/kg at harvest. The overall carbon allocation fraction to berry, shoot+trunk, and root is approximately 0.67, 0.18 and 0.15 respectively. Furthermore, in field grown vines, Greven *et al.* (2016) found the fraction of starch in root and stem biomass increased by 10% from véraison to harvest. $V_{max,stem}$ was determined for making sure the non-structural carbon concentration of stem was within the range observed in Grechi *et al.* (2007). Note, the explored value for the carbon dynamics of the stem may contain some unconsidered processes. For instance, the value of $k_{leakage}$ may contain active carbon unloading from phloem to stem for the radial growth of the internode and trunk as we try to match the overall biomass increase of the stem. Thus one should use this value with caution.

Final parameter optimization was done in sequence of carbon unloading by berry ($V_{max,berry}$, $k_{cf,}$ and C_f^*) and water uptake by berry ($L_{p,max}$, FM_{Lp}^* and k_{Lp}) through whole-plant model optimization. Parameters were optimized at whole-plant level by maximizing the sum of log-likelihood of the simulated model outputs given the observed berry dry weight and fresh weight using the random walk Markov chain Monte Carlo (MCMC) method (see the description above). Optimization was done based on the observed data of 12 leaves per cluster for both Cabernet Sauvignon and Sangiovese. The data of 3 leaves per cluster was reserved for validation. Many iterations were made to verify the stability of the parameters. After serval rounds of optimization for berry dry weight, $k_{cf,}$ and C_f^* were fixed and $V_{max,berry}$ was optimized for the dynamics of berry dry weight (Supplementary Fig. S4). After determining $V_{max,berry}$ and many rounds of optimization for berry fresh weight through adjusting $L_{p,max}$, FM_{Lp}^* and k_{Lp}, FM_{Lp}^* and k_{Lp} were fixed and $L_{p,max}$ were further optimized for the dynamics of berry fresh weight. A final round of optimization was done by optimizing both $V_{max,berry}$ and $L_{p,max}$ for the dynamics of berry dry weight and fresh weight.

Supplementary figures:

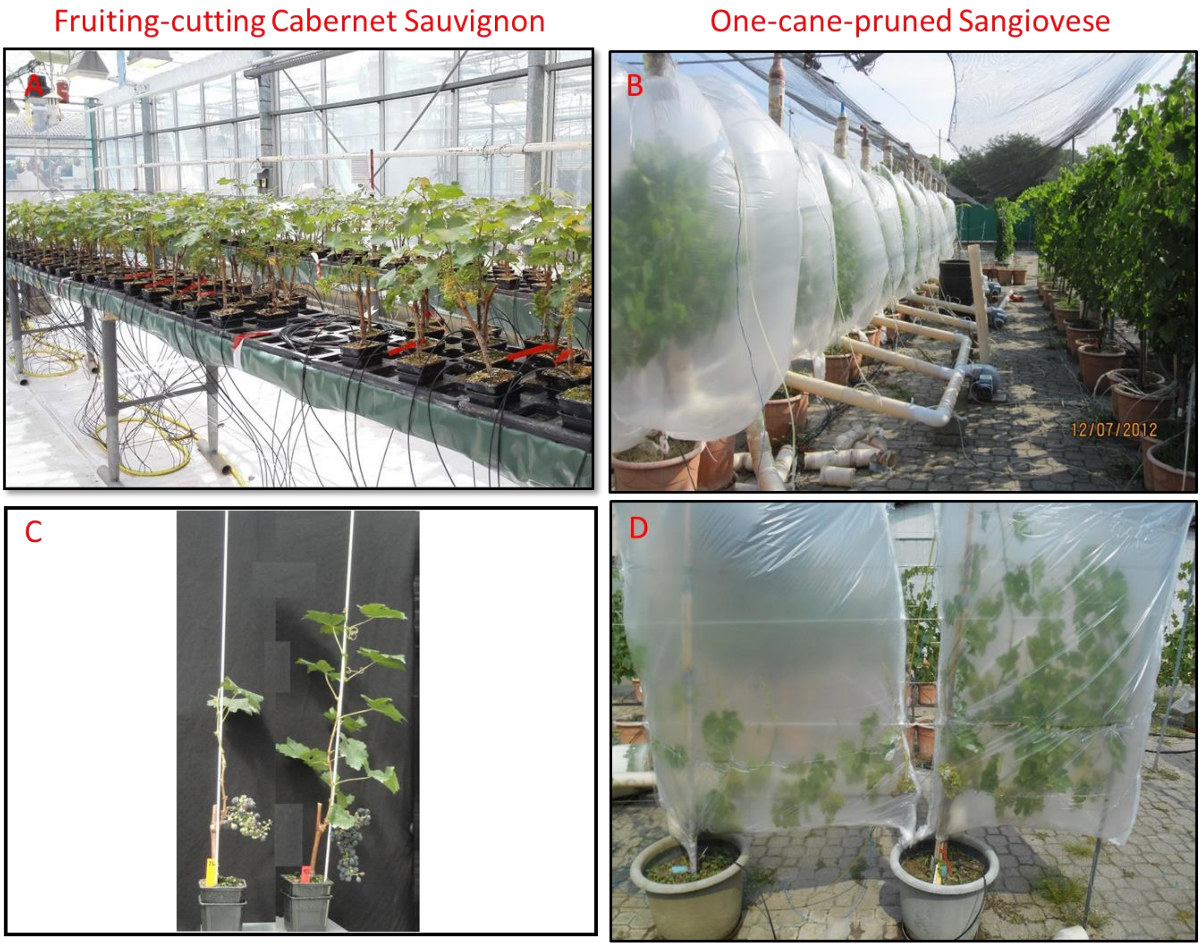

Fig. S1 Illustration of experimental condition for fruiting-cutting Cabernet Sauvignon in the greenhouse (left panels A and C), and for outdoor potted one-cane-pruned Sangiovese with the treatment of different leaf number per cluster (left panels B and D). The plastic bags around one-cane-pruned Sangiovese were used for measuring whole-plant gas exchange.

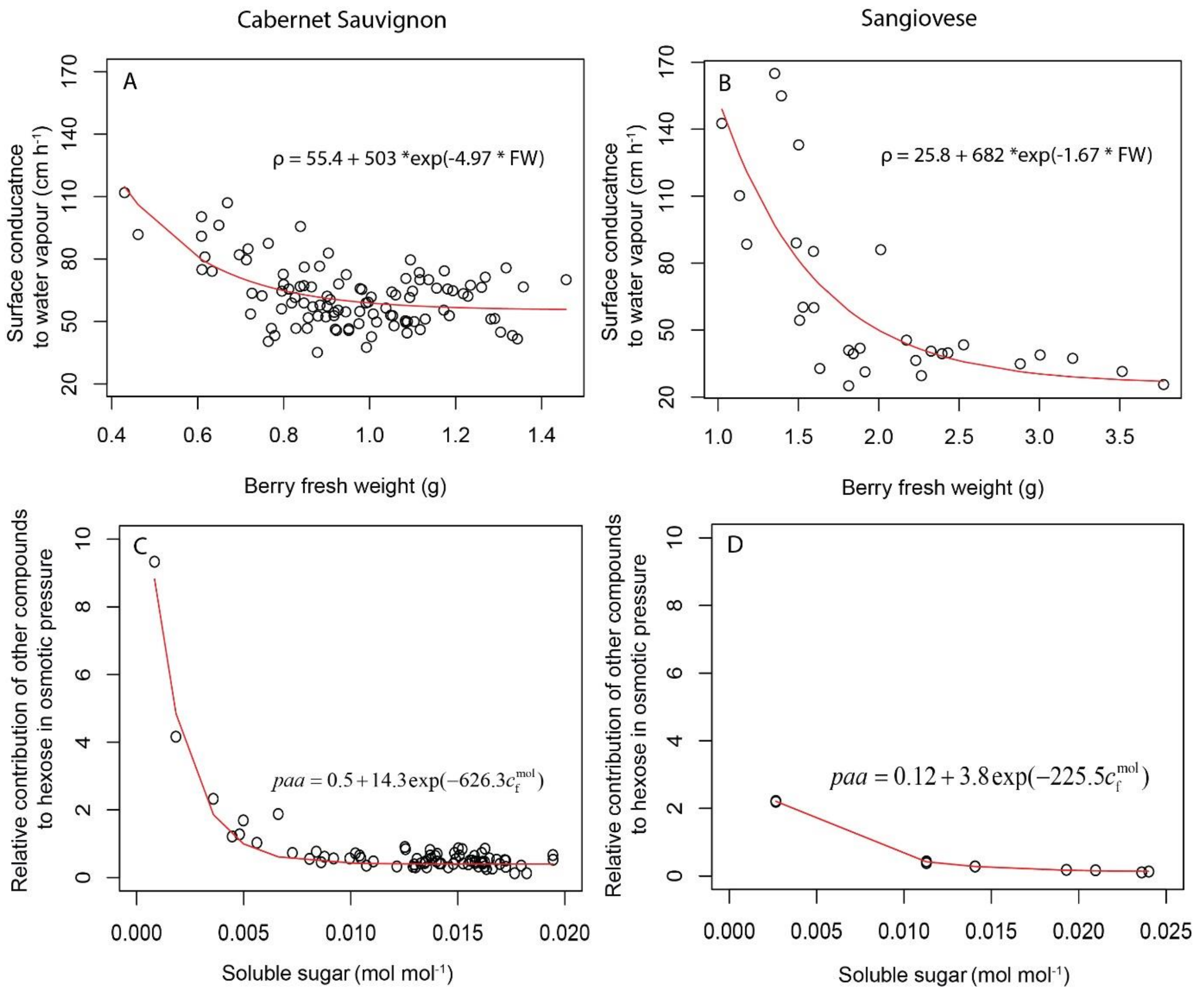

Fig. S2 Correlation between grape berry surface conductance to water vapour and berry fresh weight (upper panels) and the relative contribution of acids (for instance as amino acids and organic acids) and other compounds (for instance H^+, K^+, Ca^{2+}, Na^+, Cl^{-1} and SO_4^{-2}) to soluble sugar in the total osmotic pressure under different concentrations of soluble sugar (bottom panels). Circles were measurements at varying developmental stages and lines were the fitted curve. Left panels were data for Cabernet Sauvignon and right panels were data for Sangiovese. Berry surface conductance to water vapour decreased from 100 cm h^{-1} to 55.4 cm h^{-1} for Cabernet Sauvignon when fresh weight increased from 0.4 g to 1.4 g, and decreased from 140 cm h^{-1} to 25.8 cm h^{-1} for Sangiovese when fresh weight increased from 1.0 g to 3.5 g. The ratio between the osmotic pressure caused by acids and other soluble compounds and the osmotic pressure caused by soluble sugar in the total osmotic pressure decreased with increasing fruit hexose concentration. The ratio decreased from eight when the soluble sugar concentration was 0.001 mol mol^{-1} (~0.01 gHexose $gPulp^{-1}$, see conversion method in E10 Fig. 2 in main text) to 0.5 when the soluble sugar concentration was 0.02 mol mol^{-1} (~0.17 gHexose $gPulp^{-1}$) for Cabernet Sauvignon.

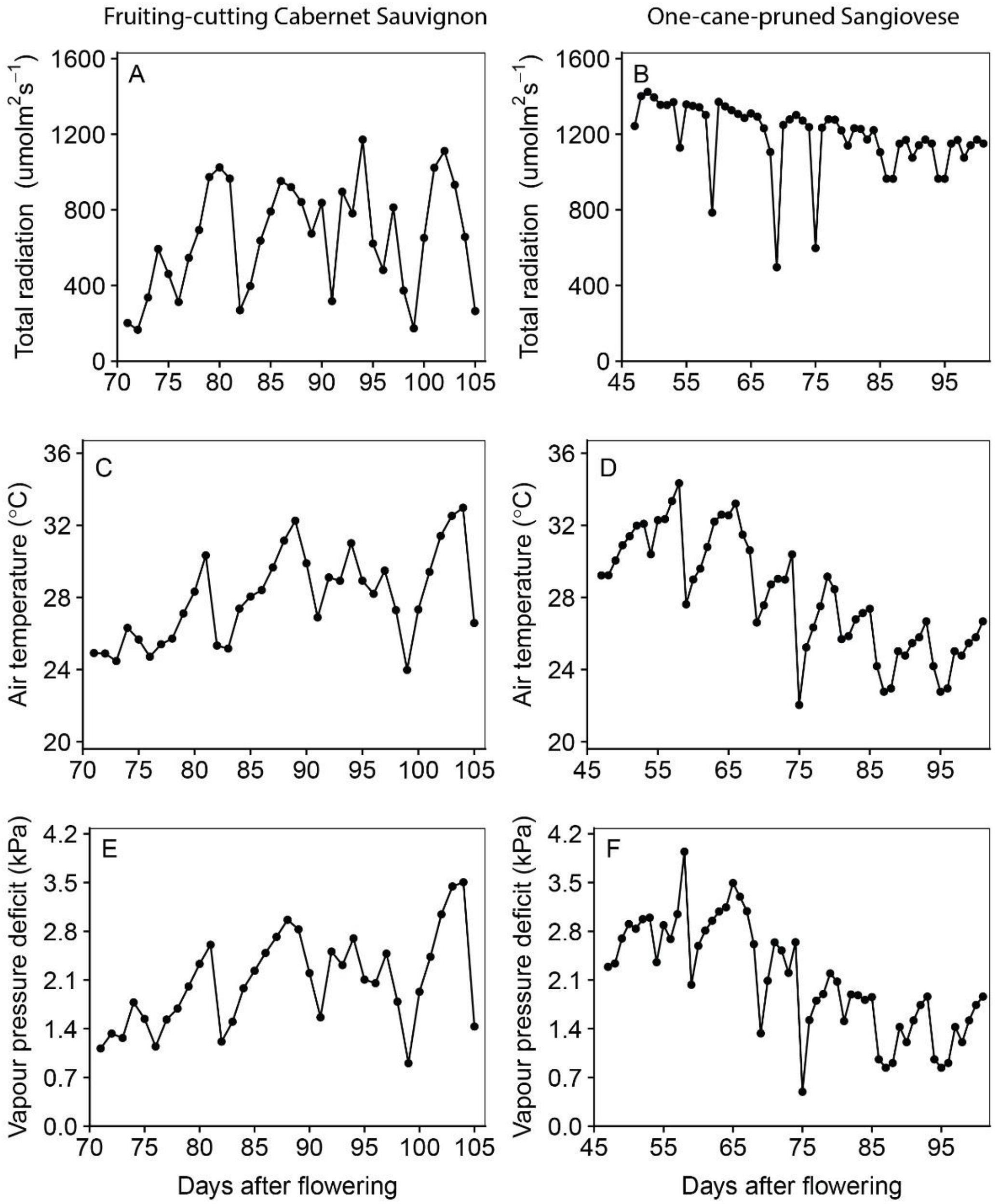

Fig. S3 Daily mean climate condition for the greenhouse fruiting-cutting Cabernet Sauvignon at Bordeaux in 2012, and for the outdoor potted one-cane-pruned cv. Sangiovese at Piacenza in 2013. As plants were regularly irrigated, soil water potential was assumed constant over time (−0.05 MPa).

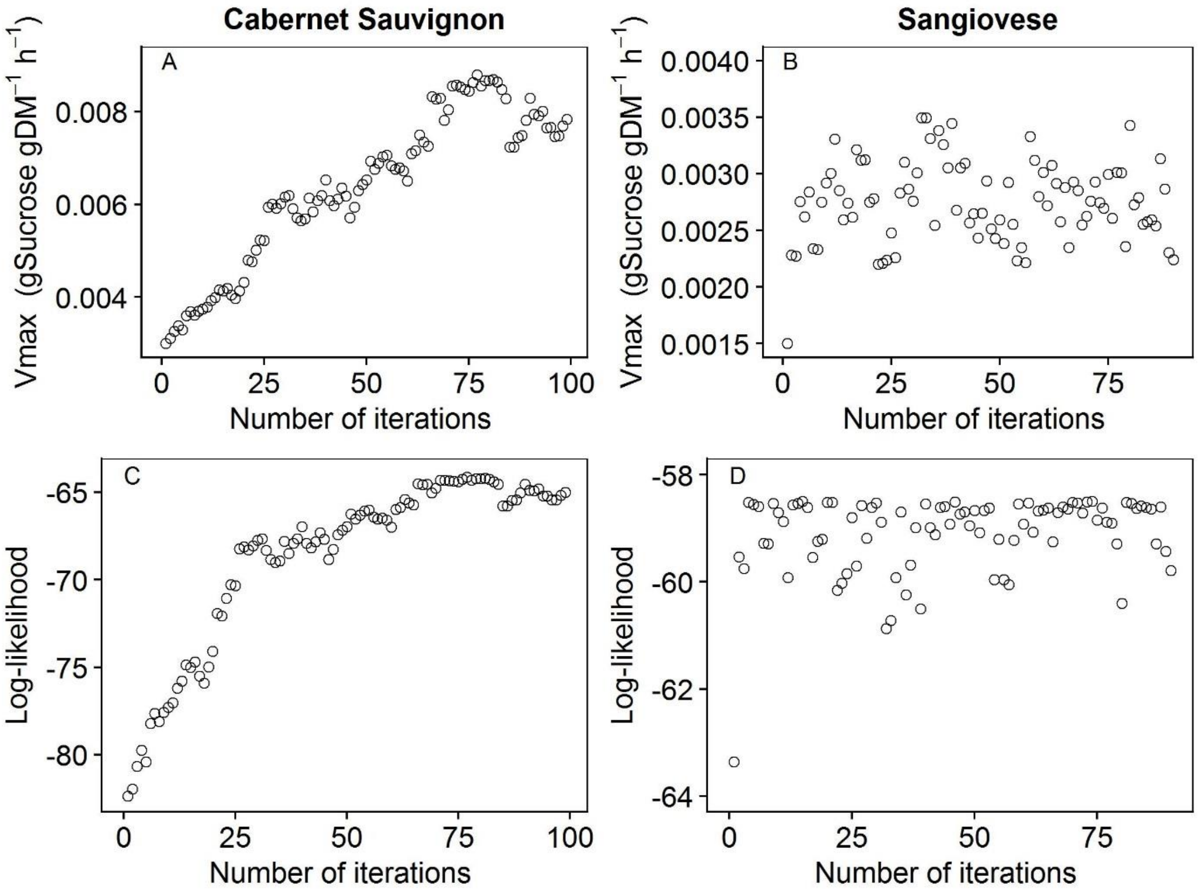

Fig. S4 Evolution of V_{max_berry} and log-likelihood during one of the random walk Markov chain Monte Carlo optimizations. Left panels are the optimizing result of for fruiting-cutting Cabernet Sauvignon with a relative low starting value for V_{max_berry}. Right panels are the optimizing result of for one-cane-pruned Sangiovese with a starting V_{max_berry} close to the final optimizing value. Optimization was done based on the dynamics of berry dry weight and fresh weight under 12L per cluster for using the dataset of Bobeica *et al.* (2015) for both Cabernet Sauvignon and Sangiovese. Mean parameter values with log-likelihood larger than -66 for Cabernet Sauvignon and with log-likelihood larger than -59 for Sangiovese were used as the final V_{max_berry} value.

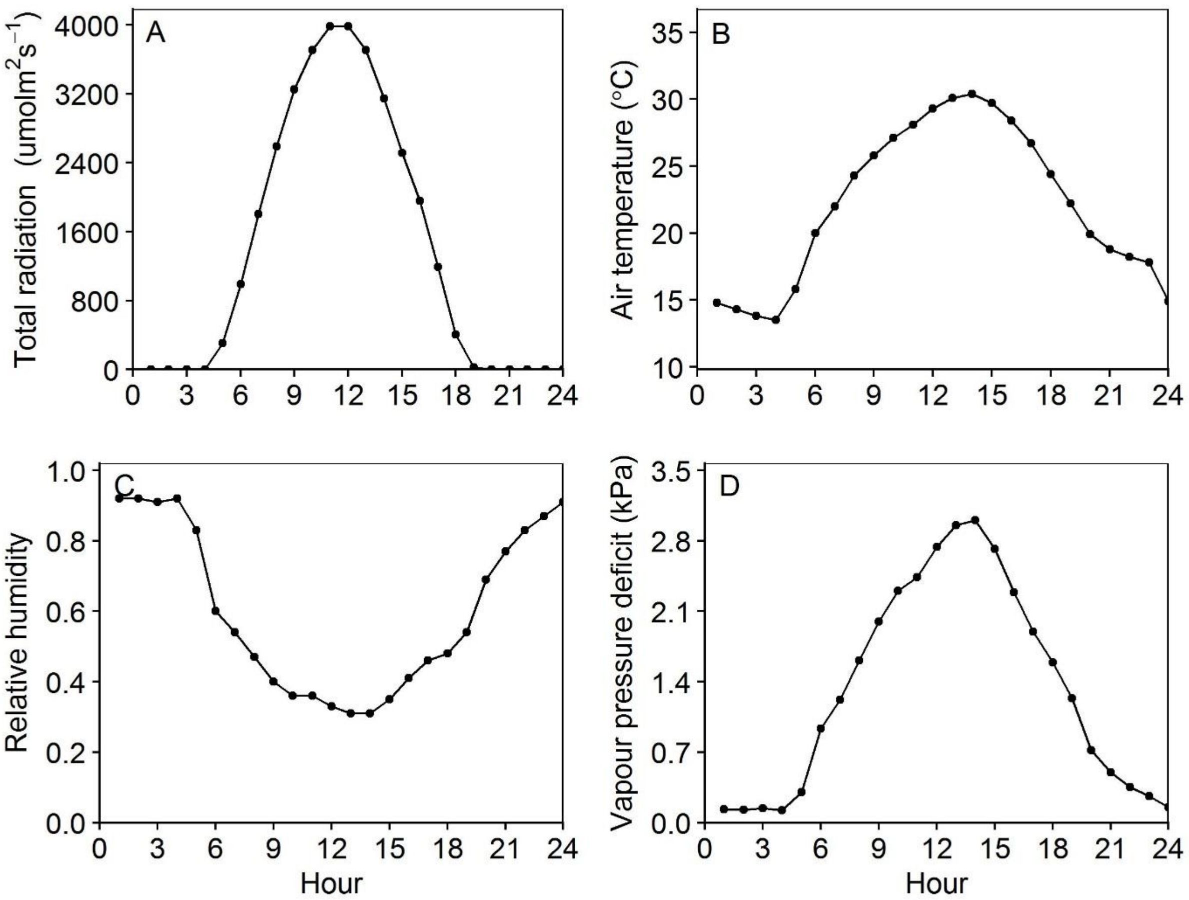

Fig. S5 diurnal dynamics of total radiation, air temperature, relative humidity and valour pressure deficit on August 7th, 2010 in the campus of INRA Bordeaux. This date is close to the date of v éraison. This diurnal data was used for the scenario simulations. Photosynthetic active radiation was about 50% of the total radiation. Vapour pressure deficit was calculated based on relative humidity and air temperature.

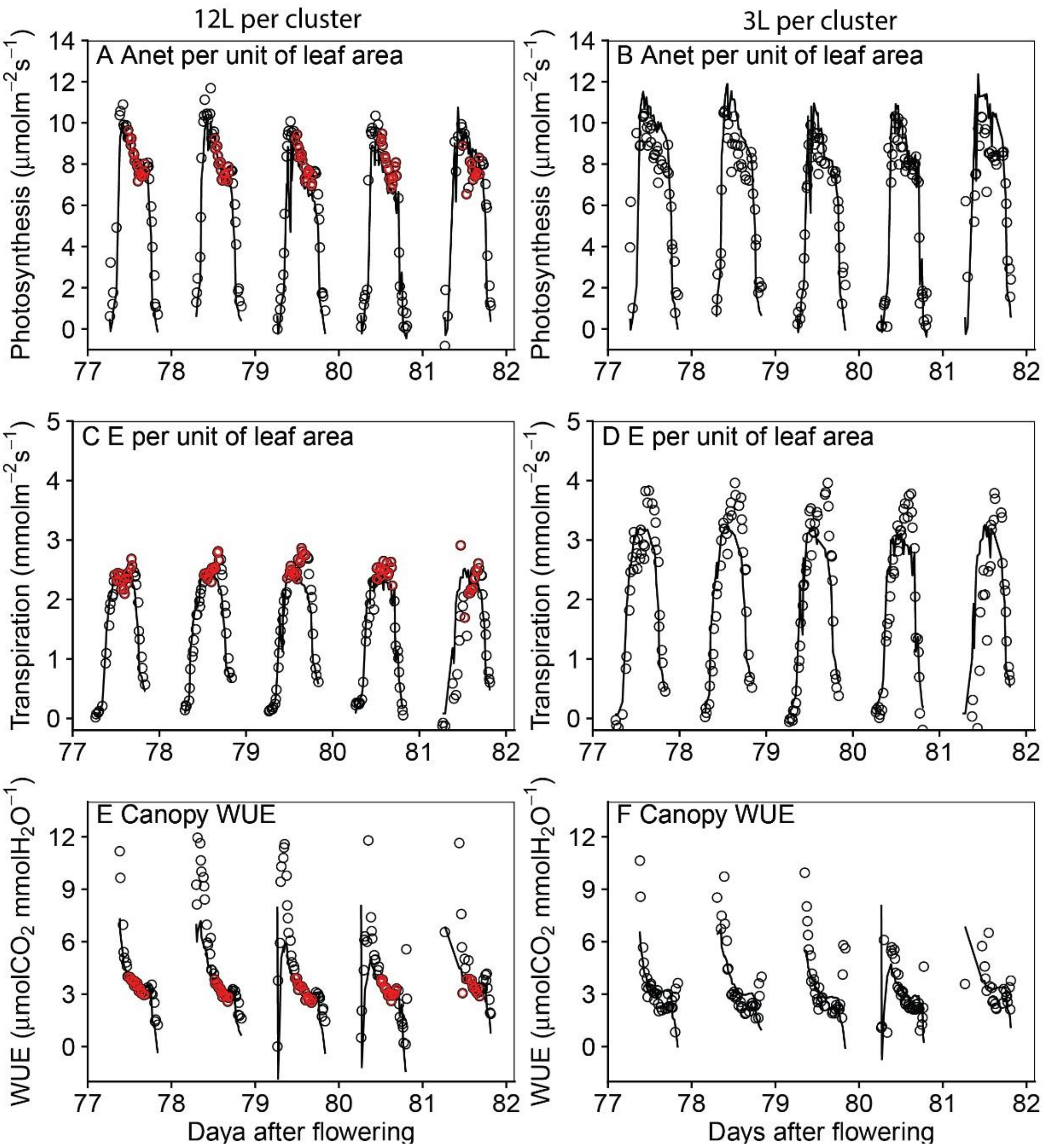

Fig. S6 Verification (left panels) and validation (right panels) of the simulated whole-canopy photosynthesis per unit of leaf area (A, B), canopy transpiration per unit of leaf area (C, D), instantaneous canopy water use efficiency (E, F) for vines with 12 leaves per cluster and 3 leaves per cluster of one-cane pruned Sangiovese. Open circles were the observed values, while lines were simulated values. The model was only optimized based on the photosynthesis, transpiration and water use efficiency data between 10 o'clock to 16 o'clock, and filtered by water use efficiency that smaller than four and larger than two. Red points in left panels were the data used for model optimization during this period, while other points were just used for model validations. The time period of day of year 228 to 232 was selected because we have the best continuous records in this period. The simulation results for the whole period of day 198 to 244 is shown in Fig. S7. The leaf area per plant for 12 leaves per cluster was 1.02 m^2, and for 3 leaves per shoot was 0.31 m^2.

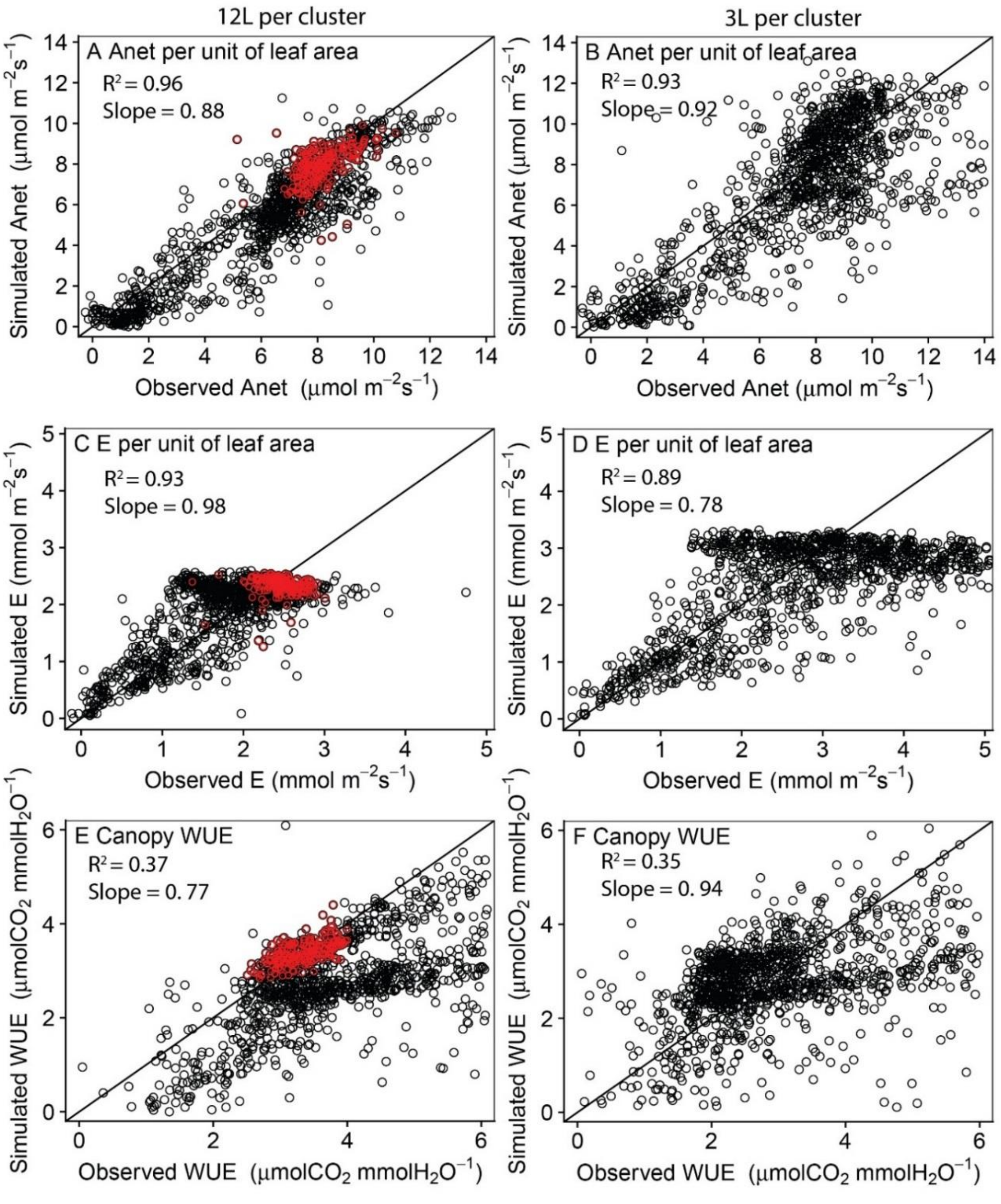

Fig. S7 Verification (left panels) and validation (right panels) of the simulated whole-canopy photosynthesis per unit of leaf area (A, B), canopy transpiration per unit of leaf area (C, D), instantaneous canopy water use efficiency (E, F) for vines with 12 leaves per cluster and 3 leaves per cluster of one-cane pruned Sangiovese. Red points are the data used for model optimization. The model was optimized based on the photosynthesis, transpiration and water use efficiency data between 10 o'clock to 16 o'clock and filtered by water use efficiency smaller than four and larger than two. Points beyond this period are validations. Only the observed data points where the water-use efficiency (WUE) is smaller than 6 umol CO_2/ mmolH_2O were shown in the figure.

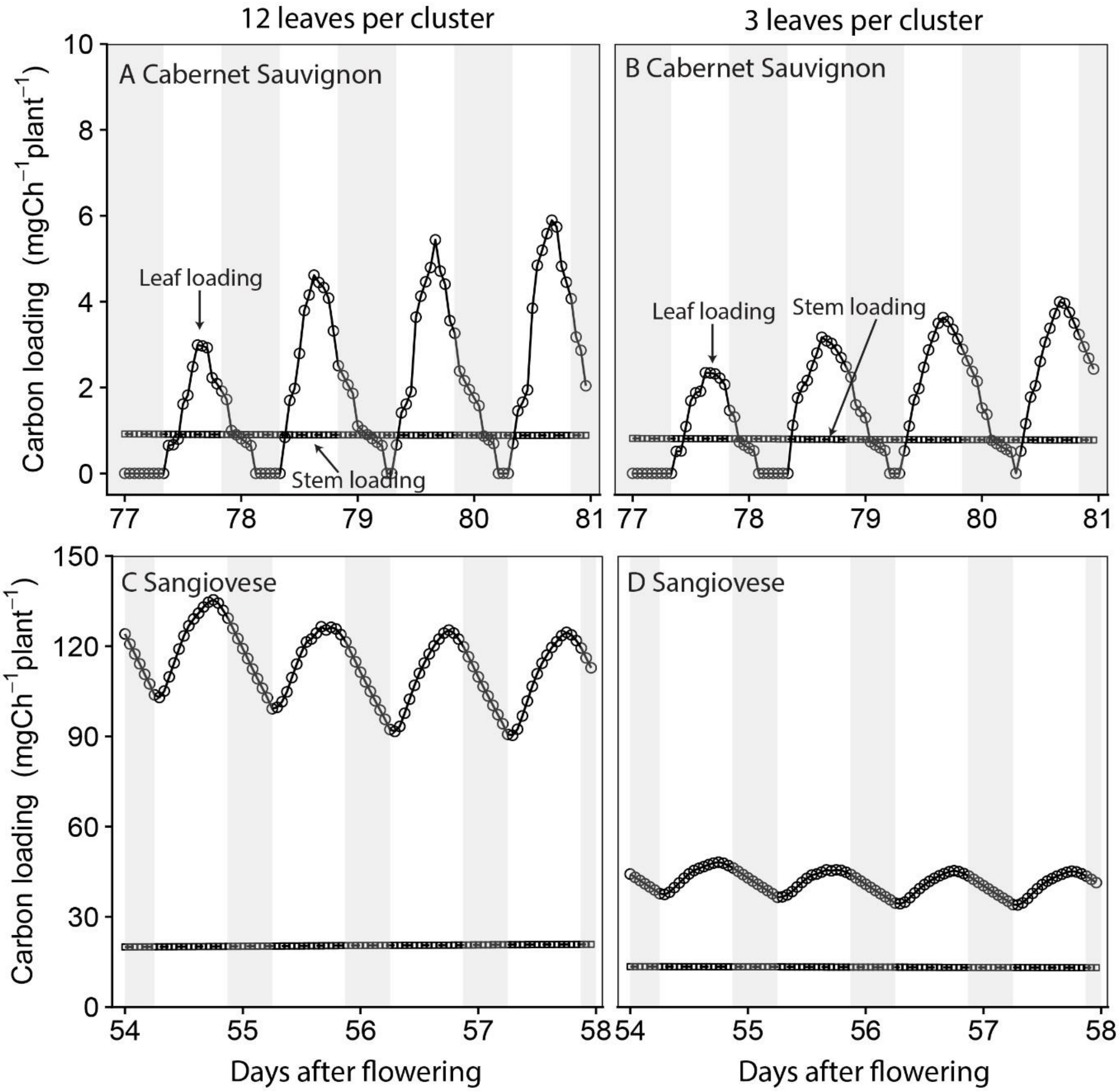

Fig. S8 Simulated diurnal whole-plant carbon loading by all leaves (circles) and stems (squares) in fruiting-cutting Carbernet Sauvignon (A and B) and one-cane-pruned Sangiovese (C and D). Left panels were 12 leaves per cluster and right panels were 3 leaves per cluster. Carbon loading from leaf to phloem gradually increased during the day, and reached the maximum around 16:00, and then decreased late in the afternoon. In one-cane-pruned Sangiovese, the carbon supply in the treatment with 12 leaves per cluster was 2.74 times of that in the treatment with 3 leaves per cluster while the leaf area ratio was 3.29. However, in fruiting-cutting Cabernet Sauvignon despite the fact that the leaf area ratio was 4.16, the model predicted that the total carbon loading by leaf in the treatment with 12 leaves per cluster was only 1.37 times of that in the treatment with 3 leaves per cluster. This contrasting results found in fruiting-cutting Cabernet Sauvignon was partly because of the high plant density used in greenhouse leading to self and mutual shading (Supplementary Fig. S1), and

partly because of the low radiation level, which was limiting leaf photosynthesis at its maximum photosynthesis rate, caused by sheltering in summer for avoiding high temperature (Supplementary Fig. S3). Shaded areas indicated the night-time period, 8 pm to 5 am.

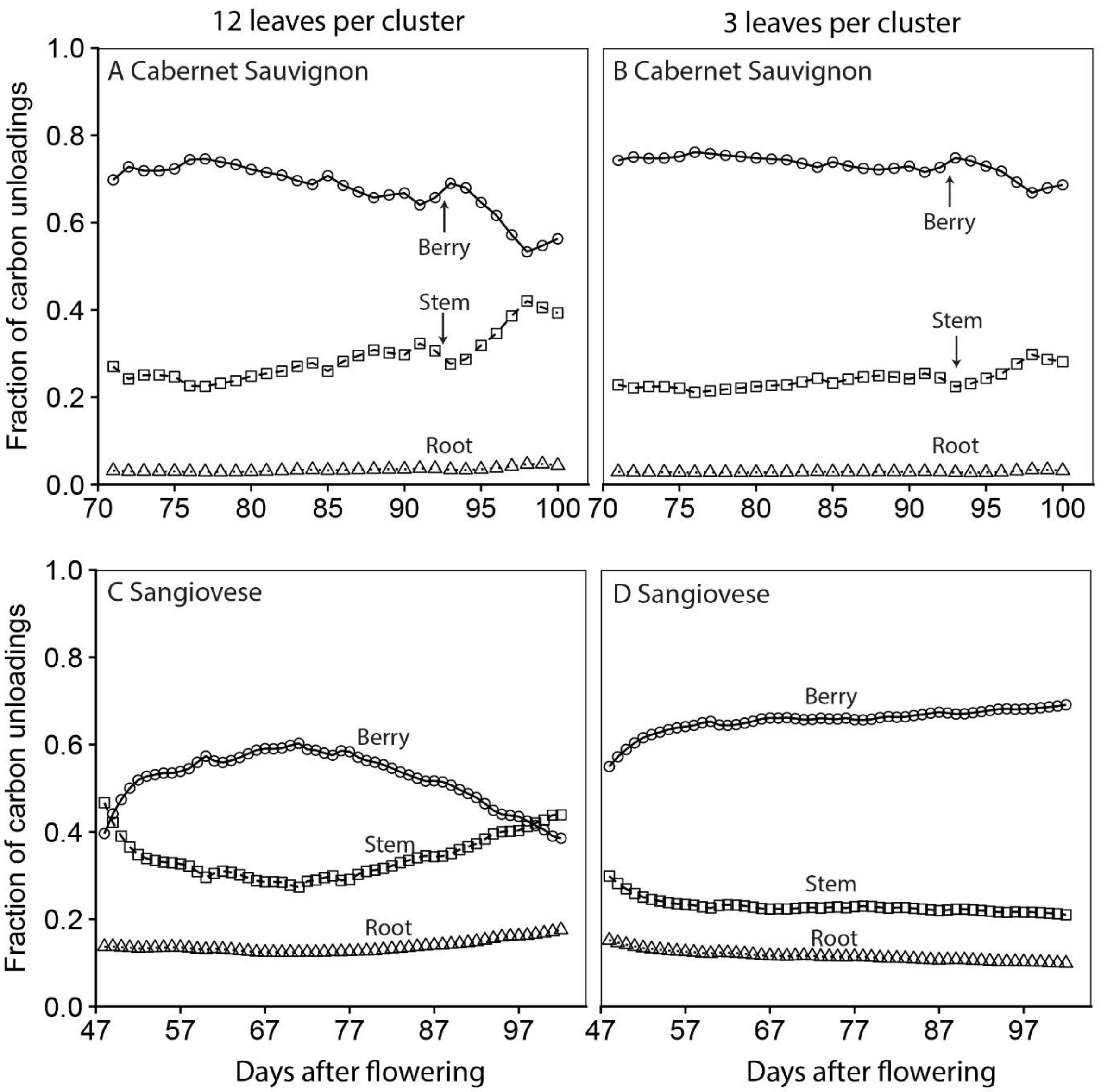

Fig. S9 Simulated mean daily fraction of carbon unloading by berries (circles), stem (squares) and root (triangles) in fruiting-cutting Cabernet Sauvignon (A and B) and one-cane-pruned Sangiovese (C and D). On average of two crop loads, leaf contributes 55.7% of the total carbon loaded in the phloem in fruiting-cutting Cabernet Sauvignon, and stem contributes 44.3% of the total carbon loaded, while in one-cane-pruned Sangiovese leaf contributes 80.1% of the total carbon loaded. Stem unloaded 26.5% of the total carbon in fruiting-cutting Cabernet Sauvignon and unloaded 28.5% in one-cane-pruned Sangiovese on average of two crop loads demonstrating the carbon leakage-reloading processes in stem (Supplementary Method S1). The fraction of carbon unloaded by berry was 67.6% in fruiting-cutting Cabernet Sauvignon with 12 leaves per cluster, and was 73.1% for 3 leaves per cluster. In one-cane-pruned Sangiovese, the fraction of carbon unloaded by berry was 52.2% for 12 leaves per

cluster and 65.5% for 3 leaves per cluster. In our model, berry sink priority was captured through the Michaelis-Menten constant (Km) values, where small values represent high priority. $K_{m,berry}$ was set as one fifth of $K_{m,root}$ (in unit of gC gH_2O^{-1}, Table 1). Thus under conditions of carbon limitation the berry would get a greater proportion of the total available carbon.

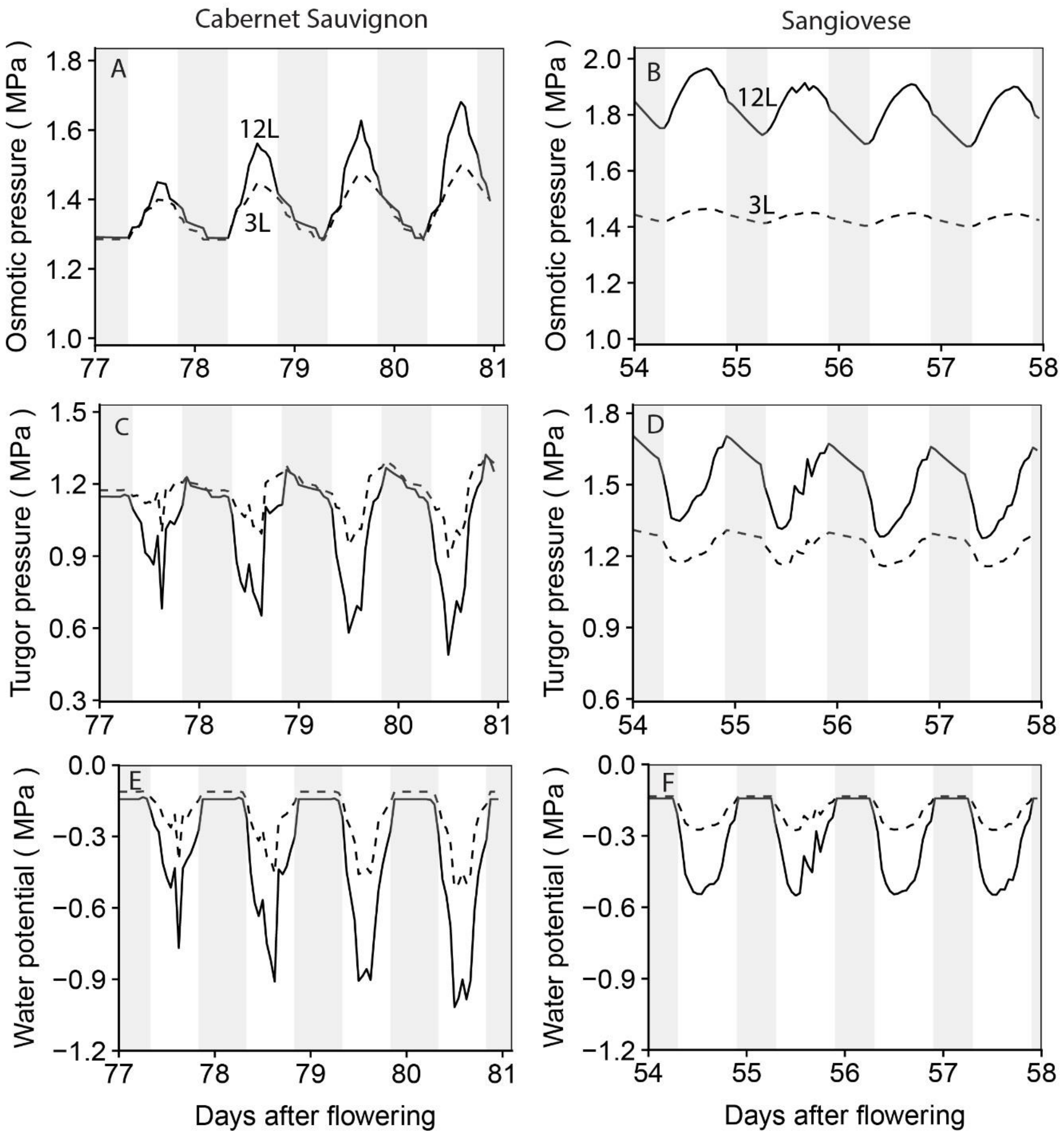

Fig. S10 Simulated diurnal changes of phloem osmotic pressure (A and B), turgor pressure (C and D) and water potential (E and F) for Cabernet Sauvignon (left panels) and Sangiovese (right panels) within a 4-day period. Solid lines represented vines with 12L per cluster, and dashed lines represented vines with 3L per cluster. Shaded areas indicated the night-time period, 8 pm to 5 am.

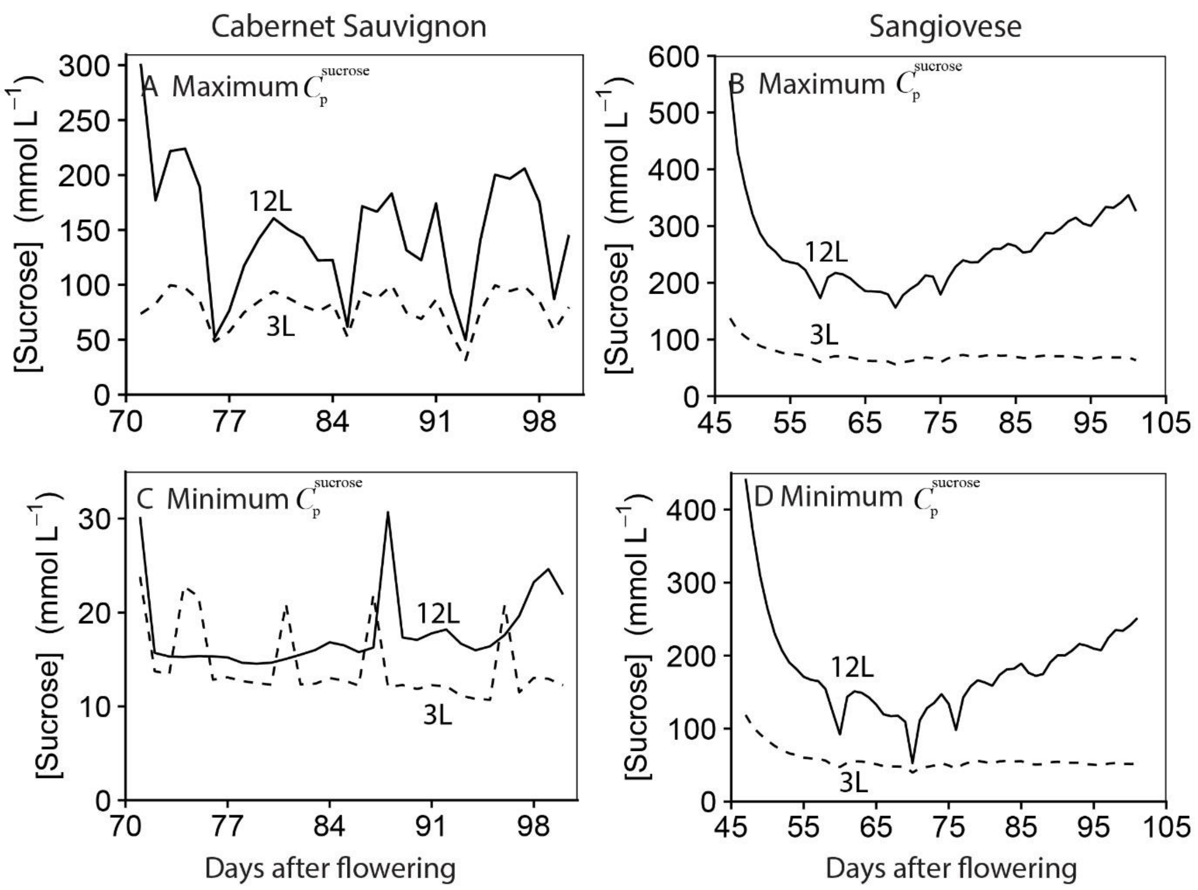

Fig. S11 Maximum daily phloem sucrose concentration (A and B), minimum daily phloem sucrose concentration (C and D) for Cabernet Sauvignon (left panels) and Sangiovese (right panels). Solid lines represented the vines with 12 leaves per cluster, and dashed lines represented vines with 3 leaves per cluster. The high phloem sucrose concentration at the start of the simulation could be because: 1) the input nonstructural carbon concentration for leaf and stem was higher than the actual condition, thus the model require some time to stabilize based on the current environmental condition; 2) berry has a lower sugar uptake capacity at the start of the simulation due to a lower dry matter.

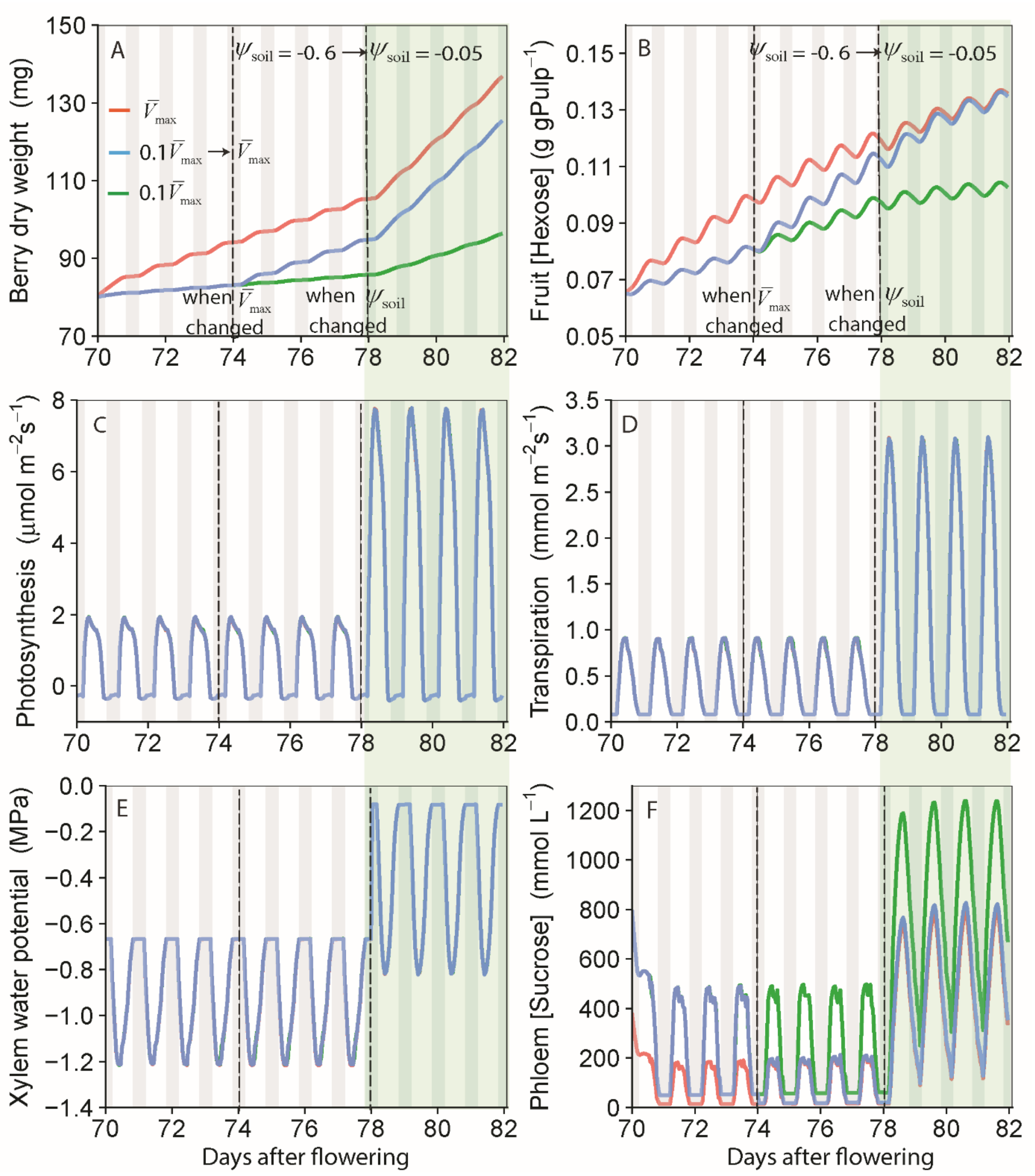

Fig. S12 The dynamics of berry dry weight (A), fruit hexose concentration (B), mean canopy photosynthesis rate (C), mean canopy transpiration rate (D), xylem water potential (E) and phloem sucrose concentration (F) under varying sugar uptake capacity ($V_{max,berry}$) with water stress for the first eight days (70 to 77 days after flowering) and well-watered for remaining four days (78 to 81 days after flowering). Solid and red lines were simulated with constant default V_{max} (Table 1). Dotted and blue lines were simulated with 0.1 V_{max} for the first four days, and then switch to V_{max} for the remaining eight days. Dashed and green lines were simulated with 0.1V_{max} throughout the whole period. Simulation was run based on the model set up for fruiting-cutting Cabernet Sauvignon system. Climatic conditions were shown in Supplementary Fig. S5. Shaded areas indicate the night-time, 8 pm to 5 am.

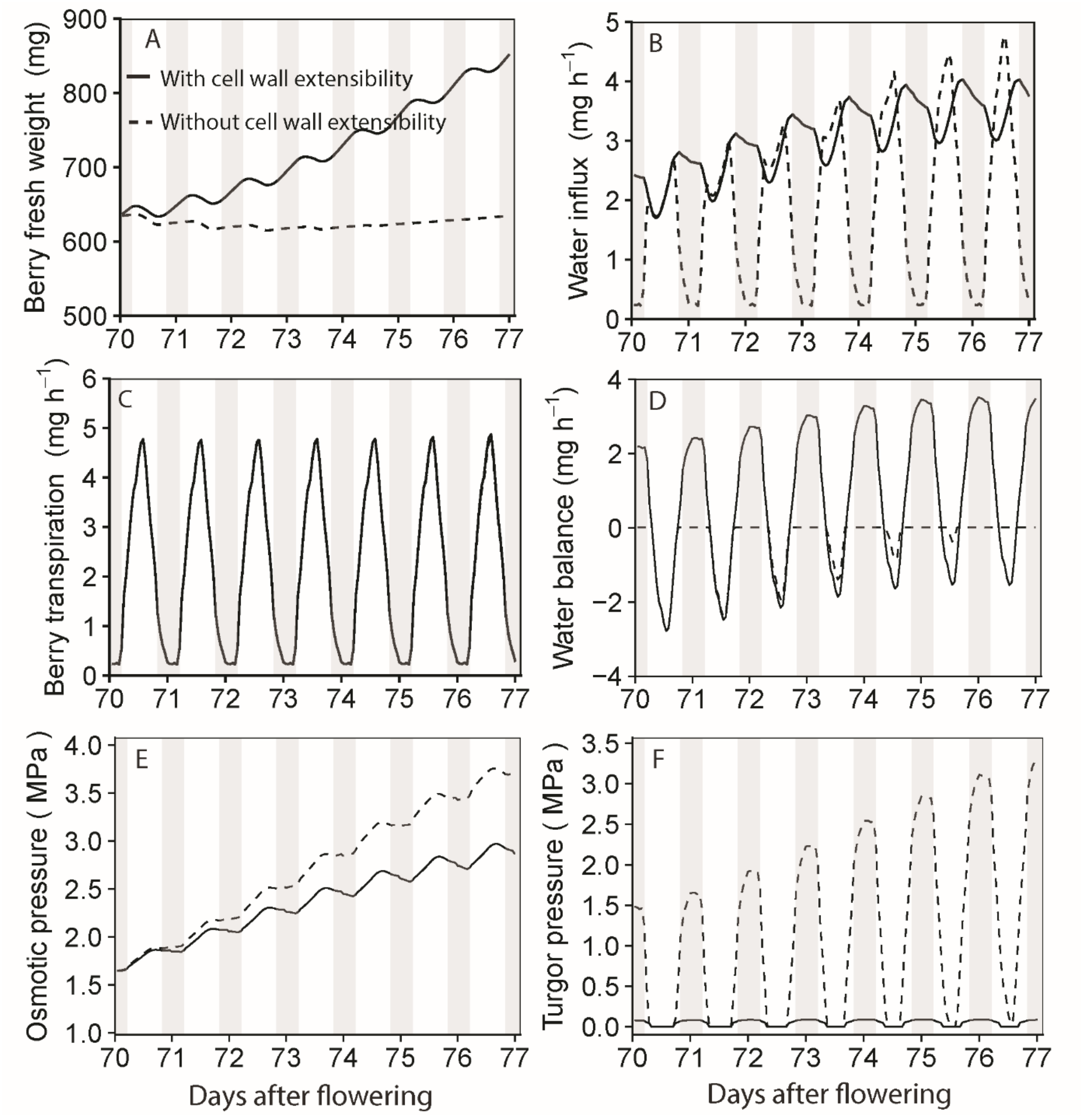

Fig. S13 The dynamics of berry fresh weight (A), water influx (B), surface transpiration (C), water balance (D), osmotic pressure (E) and turgor pressure (F) with the extensibility of cell wall being 0.1 MPa^{-1} h^{-1} (solid lines) and with no extensibility of cell wall (Ø = 0, dashed lines). Simulation was run for 7 days based on the model set up for fruiting-cutting Cabernet Sauvignon system. Climatic conditions were shown in Supplementary Fig. S5. Shaded areas indicated the night-time, 8 pm to 5 am. Reducing the cell wall extensibility to zero caused a reduction in berry FW because of a negative water balance during the day and neutral water balance during the night (A dashed line). Consequently, there was an increase in osmotic pressure (E) which increased the water influx during the day and gradually resulted in a zero water balance at daytime (D). The increase in osmotic pressure was accompanied by a raise in fruit turgor pressure (F) which pushes the cell wall to enlarge.

References:

Barillot R, Chambon C, Andrieu B. 2016. CN-Wheat, a functional–structural model of carbon and nitrogen metabolism in wheat culms after anthesis. I. Model description. *Annals of Botany* **118**, 997-1013 doi: 10.1093/aob/mcw143.

Bobeica N, Poni S, Hilbert G, Renaud C, Gomès E, Delrot S, Dai Z. 2015. Differential responses of sugar, organic acids and anthocyanins to source-sink modulation in Cabernet Sauvignon and Sangiovese grapevines. *Frontiers in plant science* **6**, 382-382 doi: 10.3389/fpls.2015.00382.

Buwalda J. 1993. The carbon costs of root systems of perennial fruit crops. *Environmental and Experimental Botany* **33**, 131-140.

Castelan-Estrada M, Vivin P, Gaudillere JP. 2002. Allometric relationships to estimate seasonal above-ground vegetative and reproductive biomass of Vitis vinifera L. *Annals of Botany* **89**, 401-408 doi: 10.1093/aob/mcf059.

Castellarin SD, Gambetta GA, Wada H, Krasnow MN, Cramer GR, Peterlunger E, Shackel KA, Matthews MA. 2016. Characterization of major ripening events during softening in grape: Turgor, sugar accumulation, abscisic acid metabolism, colour development, and their relationship with growth. *Journal of Experimental Botany* **67**, 709-722 doi: 10.1093/jxb/erv483.

Cieslak M, Seleznyova AN, Hanan J. 2011. A functional-structural kiwifruit vine model integrating architecture, carbon dynamics and effects of the environment. *Annals of Botany* **107**, 747-764 doi: 10.1093/aob/mcq180.

De Vries FP, Van Laar H. 1982. Simulation of growth processes and the model BACROS. In: De Vries FP, Van Laar H, eds. *Simulation of plant growth and crop production*. Wageningen, The Netherlands: Pudoc, 114-135.

Fishman S, Génard M. 1998. A biophysical model of fruit growth: simulation of seasonal and dirunal dynamics of mass. *Plant, cell and environment* **21**, 739-752.

Grechi I, Vivin P, Hilbert G, Milin S, Robert T, Gaudillère J-P. 2007. Effect of light and nitrogen supply on internal C: N balance and control of root-to-shoot biomass allocation in grapevine. *Environmental and Experimental Botany* **59**, 139-149.

Greven MM, Neal SM, Tustin DS, Boldingh H, Bennett J, Vasconcelos MC. 2016. Effect of Postharvest Defoliation on Carbon and Nitrogen Resources of High-Yielding Sauvignon blanc Grapevines. *American Journal of Enology and Viticulture* **67**, 315-326 doi: 10.5344/ajev.2016.15081.

Janssens IA, Sampson DA, Curiel-Yuste J, Carrara A, Ceulemans R. 2002. The carbon cost of fine root turnover in a Scots pine forest. *Forest Ecology and Management* **168**, 231-240 doi: http://dx.doi.org/10.1016/S0378-1127(01)00755-1.

Krasnow M, Matthews M, Shackel K. 2008. Evidence for substantial maintenance of membrane integrity and cell viability in normally developing grape (Vitis vinifera L.) berries throughout development. *Journal of Experimental Botany* **59**, 849-859.

Matthews MA, Thomas T, Shackel K. 2009. Fruit ripening in Vitis vinifera L.: possible relation of veraison to turgor and berry softening. *Australian Journal of Grape and Wine Research* **15**, 278-283.

Ollat N, Gaudillere JP. 1998. The effect of limiting leaf area during stage I of berry growth on development and composition of berries of *Vitis vinifera* L. cv. Cabernet Sauvignon. *American Journal of Enology and Viticulture* **49**, 251-258.

Patrick JW, Offler CE. 1996. Post-sieve element transport of photoassimilates in sink regions. *Journal of Experimental Botany* **47**, 1165-1177.

Patrick JW, Zhang W, Tyerman SD, Offler CE, Walker NA. 2001. Role of membrane transport in phloem translocation of assimilates and water. *Functional Plant Biology* **28**, 697-709 doi: https://doi.org/10.1071/PP01023.

Poni S, Tagliavini M, Neri D, Scudellari D, Toselli M. 1992. Influence of root pruning and water stress on growth and physiological factors of potted apple, grape, peach and pear trees. *Scientia Horticulturae* **52**, 223-236.

Rossouw GC, Smith JP, Barril C, Deloire A, Holzapfel BP. 2017. Carbohydrate distribution during berry ripening of potted grapevines: impact of water availability and leaf-to-fruit ratio. *Scientia Horticulturae* **216**, 215-225.

Thomas TR, Matthews MA, Shackel KA. 2006. Direct in situ measurement of cell turgor in grape (Vitis vinifera L.) berries during development and in response to plant water deficits. *Plant, cell and environment* **29**, 993-1001.

Thomas TR, Shackel KA, Matthews MA. 2008. Mesocarp cell turgor in Vitis vinifera L. berries throughout development and its relation to firmness, growth, and the onset of ripening. *Planta* **228**, 1067.

Van Bel A. 1996. Interaction between sieve element and companion cell and the consequences for photoassimilate distribution. Two structural hardware frames with associated physiological software packages in dicotyledons? *Journal of Experimental Botany* **47**, 1129-1129.

Zhu J, Dai Z, Vivin P, Gambetta GA, Henke M, Peccoux A, Ollat N, Delrot S. 2018. A 3-D functional–structural grapevine model that couples the dynamics of water transport with leaf gas exchange. *Annals of Botany* **121**, 833-848 doi: 10.1093/aob/mcx141.
